# Divergent strategies in cranial biomechanics and feeding ecology of the ankylosaurian dinosaurs

**DOI:** 10.1038/s41598-023-45444-1

**Published:** 2023-10-25

**Authors:** Antonio Ballell, Bohao Mai, Michael J. Benton

**Affiliations:** Bristol Palaeobiology Group, School of Earth Sciences, Life Sciences Building, University of Bristol, Tyndall Avenue, Bristol, BS8 1TQ UK

**Keywords:** Palaeontology, Biomechanics

## Abstract

Ankylosaurs were important megaherbivores of Jurassic and Cretaceous ecosystems. Their distinctive craniodental anatomy and mechanics differentiated them from coexisting hadrosaurs and ceratopsians, and morphological evidence suggests dietary niche partitioning between sympatric ankylosaurids and nodosaurids. Here, we investigate the skull biomechanics of ankylosaurs relative to feeding function. First, we compare feeding functional performance between nodosaurids and ankylosaurids applying finite element analysis and lever mechanics to the skulls of *Panoplosaurus mirus* (Nodosauridae) and *Euoplocephalus tutus* (Ankylosauridae). We also compare jaw performance across a wider sample of ankylosaurs through lever mechanics and phylogenetic comparative methods. Mandibular stress levels are higher in *Euoplocephalus*, supporting the view that *Panoplosaurus* consumed tougher foodstuffs. Bite force and mechanical advantage (MA) estimates indicate that *Panoplosaurus* had a relatively more forceful and efficient bite than *Euoplocephalus*. There is little support for a role of the secondary palate in resisting feeding loads in the two ankylosaur clades. Several ankylosaurs converged on similar jaw mechanics, while some nodosaurids specialised towards high MA and some ankylosaurids evolved low MA jaws. Our study supports the hypothesis that ankylosaurs partitioned dietary niches in Late Cretaceous ecosystems and reveals that the two main ankylosaur clades evolved divergent evolutionary pathways in skull biomechanics and feeding habits.

## Introduction

The Ankylosauria were quadrupedal, large, herbivorous ornithischian dinosaurs from the Middle Jurassic to the end of the Cretaceous^1–3^. They are best known for the extensive armour of osteoderms covering their backs and sides, and their profusely ornamented skulls. They comprise two major subclades, the Nodosauridae with plain tails and the Ankylosauridae with tail clubs formed from fused terminal caudal vertebrae and osteoderms. For over 100 million years, ankylosaurs were key components of Mesozoic terrestrial ecosystems, but were particularly successful in the Late Cretaceous of North America and Mongolia^1–4^.

The unique body plan of ankylosaurs has prompted efforts to infer the functional significance of their bizarre characteristics and reconstruct their palaeobiology. Neuroanatomical studies have revealed an interesting morphological diversity of endocranial structures among ankylosaurs, which suggest an array of different sensory capabilities and behaviours, and that ankylosaurids were more active and socially complex than nodosaurids^5–9^. The ankylosaur appendicular musculoskeletal system was also unique among ornithischians, related to the acquisition of their specific mode of graviportal, quadrupedal posture and locomotion^10–12^. The extensive dermal armour of ankylosaurs and its possible defensive, display and thermoregulatory functions have also attracted attention^13^, as well as the evolution of tail weaponry in the clade^14,15^, and how tail clubs might have been used in late-diverging ankylosaurids for interspecific combat or defence against predation^16^.

Other aspects of the ankylosaur body plan and their functional value remain poorly understood. One such characteristic is the secondary palate, a structure that was independently acquired in several species of ankylosaurids and nodosaurids, being a more complex, bipartite structure in the former^1,17,18^. The osseous secondary palate of extant mammals and crocodilians separates the nasal and oral cavities^19^, and is implicated in bracing the skull against loads^20,21^. In groups of theropods with a secondary palate, this structure might have been important in resisting bending of the snout^22^. A similar mechanical role has been proposed for the secondary palate of ankylosaurs, specifically related to resisting forces derived from complex jaw mechanics^1,17^, although this hypothesis has not yet been tested.

Ankylosaurs were traditionally considered as unsophisticated herbivores capable only of processing soft and non-resistant foodstuffs via simple, vertically (i.e., orthally) directed jaw movements^4,23,24^. However, dental wear analyses and other functional morphology indices indicate that more complex jaw mechanisms involving orthal and palinal (i.e., in anteroposterior direction) power strokes during jaw occlusion were independently acquired by derived ankylosaurids and nodosaurids, painting a complex picture of jaw mechanics and feeding ecology among ankylosaurs^4,25,26^. Multiple lines of evidence suggest that ankylosaurs partitioned dietary niches with sympatric megaherbivore dinosaurs—hadrosaurs and ceratopsians—in Late Cretaceous ecosystems in North America^24,27–30^. Furthermore, interspecific niche partitioning among sympatric ankylosaurs and nodosaurs has been suggested based on differences in dentition morphology and jaw mechanics^4,29^. Specifically, nodosaurids are thought to have evolved more efficient jaw mechanisms that allowed them to consume more resistant plant materials than the coeval ankylosaurids^4,29^.

**Figure 1:**
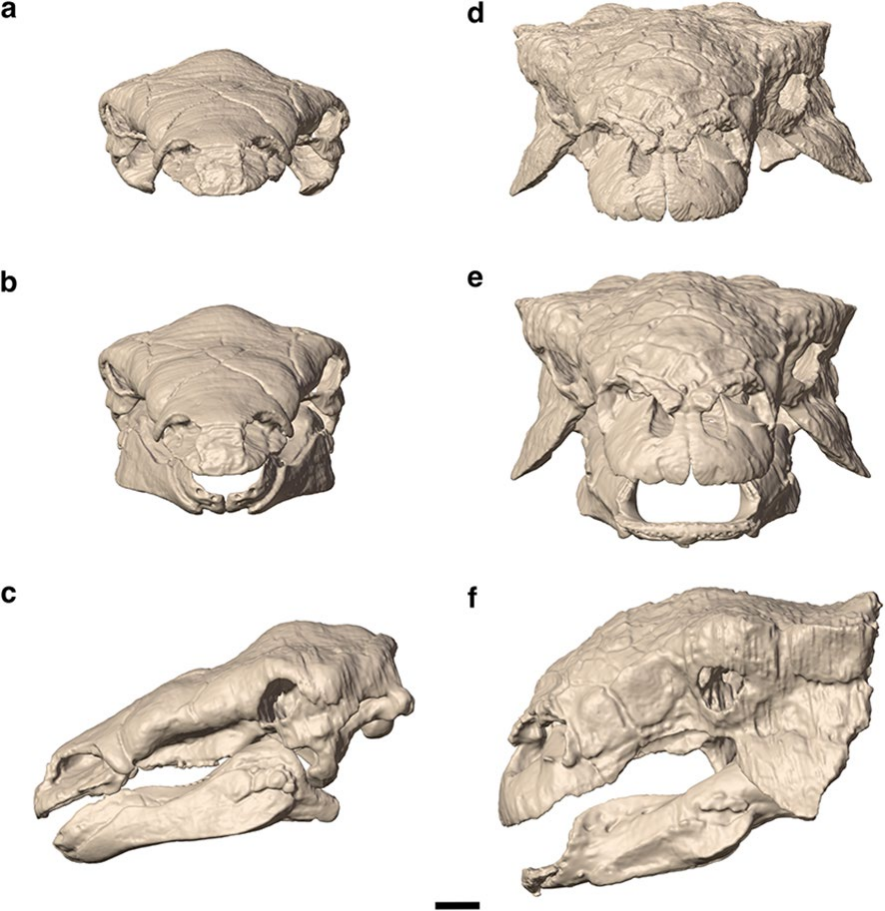
Digital 3D models of the skulls of *Panoplosaurus mirus* (**a**–**c**) and *Euoplocephalus tutus* (**d**–**f**). (**a, d**) original skull models of *Panoplosaurus mirus* (ROM 1215) and *Euoplocephalus tutus* (AMNH 5405) in anterior view. Retrodeformed, articulated skull models in anterior (**b**, **e**) and left lateral (**c**, **f**) views. Scale bar equals 5 cm.

**Figure 2:**
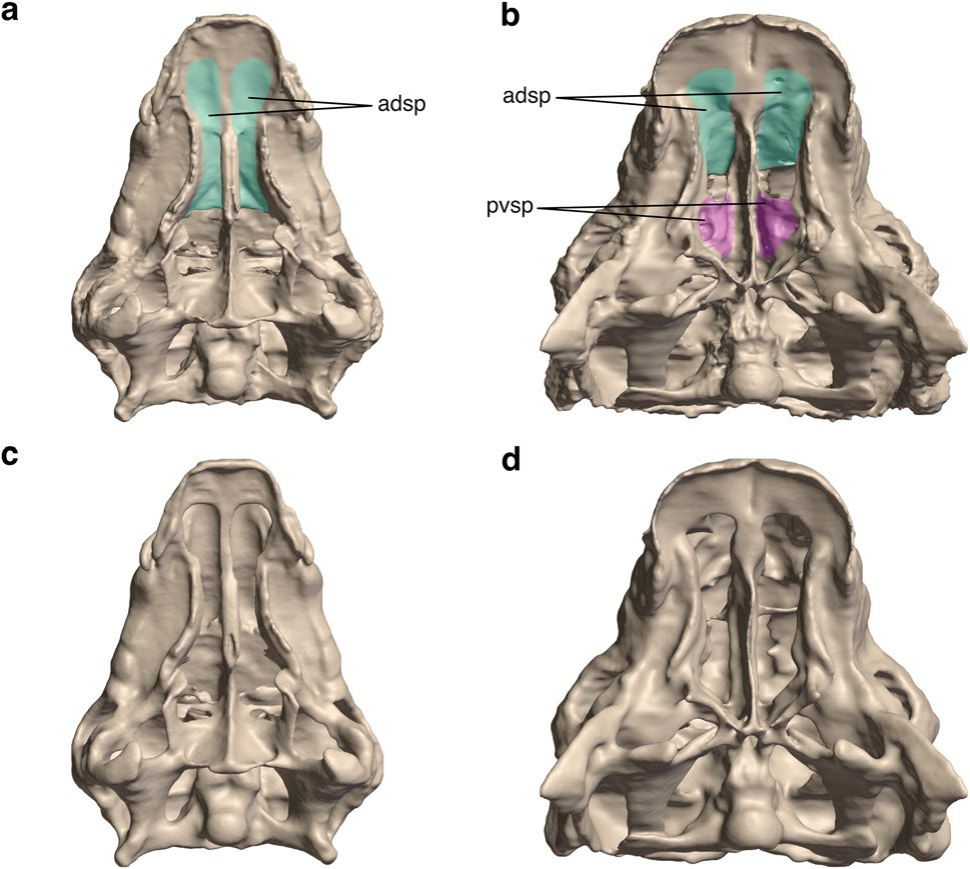
Original and hypothetical 3D models of the crania of *Panoplosaurus mirus* (**a**, **c**) and *Euoplocephalus tutus* (**b**, **d**) in palatal view. (**a**, **b**) Original crania with intact secondary palates. (**c**, **d**) Hypothetical models with digitally removed secondary palates. *Panoplosaurus* lacks the single part (anterodorsal) of its secondary palate (**c**) and *Euoplocephalus* lacks both the anterodorsal and posteroventral parts of its secondary palate (**d**). Abbreviations: adsp, anterodorsal secondary palate; pvsp, posteroventral secondary palate.

Here, we present the first functional assessment of ankylosaur cranial biomechanics, using both finite element analysis (FEA) based on digitally reconstructed jaw adductor musculatures and biting performance proxies derived from lever mechanics. FEA is a well-established computational engineering technique to test stress and strain of structures under loads^31^ and has been widely applied to living and extinct animals, especially to skulls^32–39^. Our main goal is to reconstruct the relative biomechanical behaviour of the skull during feeding in ankylosaurs, shedding light on the functional value of specific ankylosaurian traits (i.e., skull ornamentation and the secondary palate), and investigate the diversity in jaw function among different species. In particular, we compare skull biomechanical performance under feeding-related loading in two Late Cretaceous sympatric ankylosaur taxa, *Panoplosaurus mirus* (Nodosauridae) and *Euoplocephalus tutus* (Ankylosauridae) with distinct cranial and mandibular morphologies (Fig. 1) and test the possible structural role of the secondary palates of these taxa (Fig. 2). We also explore the evolution of mandibular mechanical advantage (MA) in a wider sample of ankylosaurs to test whether nodosaurids and ankylosaurids evolved divergent jaw mechanics associated with dietary specialisation. Our study reveals clear differences in skull biomechanics between *Euoplocephalus* and *Panoplosaurus* and the divergent trends in mandibular function between the two major lineages of ankylosaurs.

## Results

### Jaw adductor musculature

The morphology of the adductor chamber in ankylosaurs is unusual in the closure of the temporal roof and the presence of the postocular shelf^1,18^. This suggests that the jaw adductors were also unusual in their arrangement. The lack of clear osteological correlates for the attachment sites of multiple muscles are a source of some uncertainty in the reconstruction, and we explain our decisions below.

#### m. adductor mandibulae externus (mAME) complex

**Figure 3:**
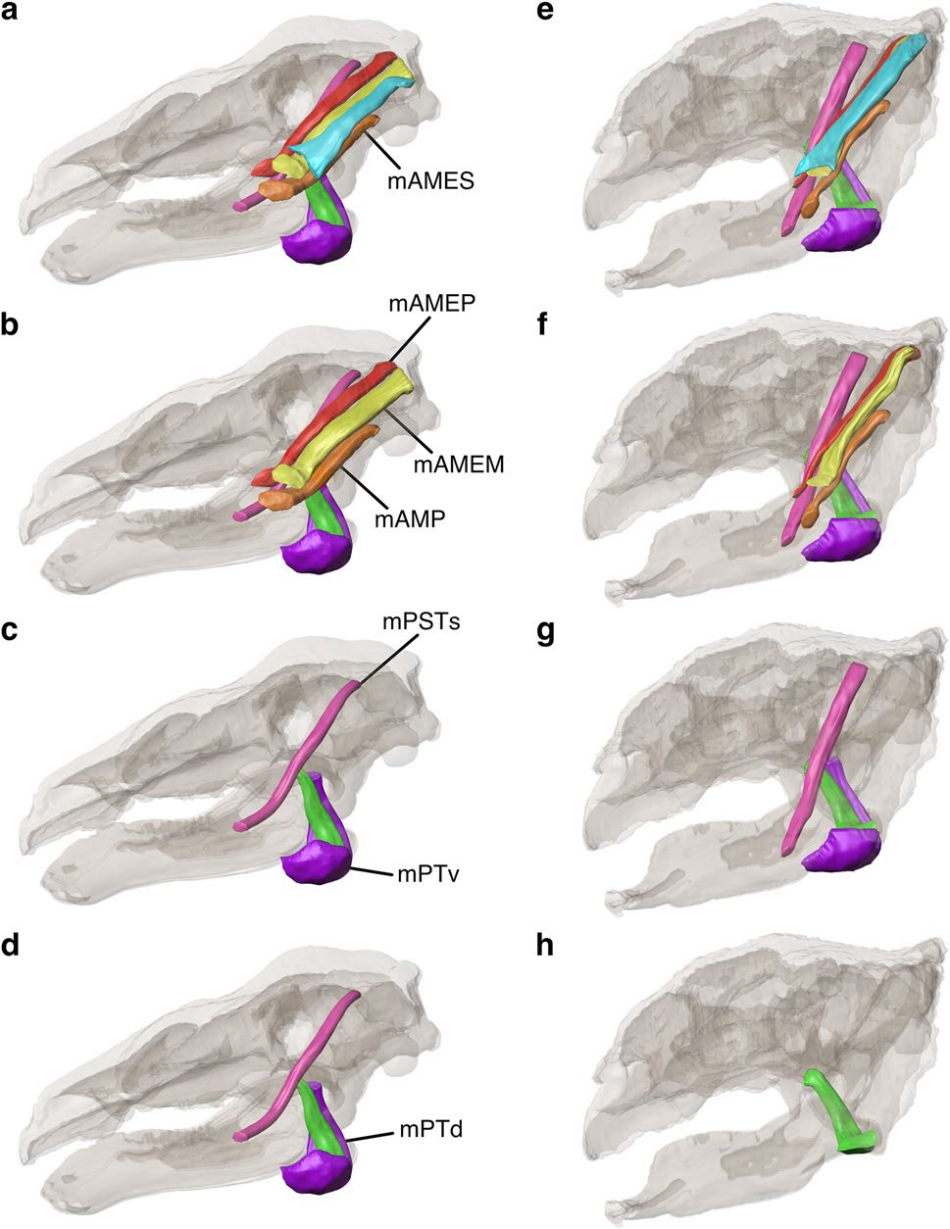
Digitally reconstructed jaw adductor muscles of *Panoplosaurus mirus* (**a**–**d**) and *Euoplocephalus tutus* (**e**–**h**) with skulls shown in left lateral view. (**a**, **e**) all reconstructed muscles. (**b**, **f**) mAMES removed. (**c**, **g**) mAMEM, mAMEP and mAMP removed. (**d**, **h**) mPSTs and mPTv removed.

The mAME muscle complex, which includes the mAMES, the mAMEM and the mAMEP, occupies most of the adductor chamber in both *Panoplosaurus* and *Euoplocephalus* (Fig. 3a, e). Due to the posterior displacement of the temporal region, the adductor chamber is angled in both species and the mAME complex travels from its origin in the temporal region posterodorsally to the exit of the adductor chamber anteroventrally. The angle offset of the mAME complex from the vertical plane is more pronounced in *Panoplosaurus* than *Euoplocephalus* as the quadrate and the adductor chamber of the former are angled more horizontally (Fig. 3). Rostrally, the extent of the mAME muscles is restricted by the postocular shelf (Fig. S1d, h), a bony structure formed by the postorbital and emerging from the skull roof posterior to the optic region^18,29,40^. While the postocular shelf only descends a short distance from the skull roof in *Panoplosaurus*, it forms a bony wall in *Euoplocephalus* that entirely partitions the mAME complex from the orbital cavity as well as from the mPST. In either case, the postocular shelf largely defines the muscle path of the mAME complex within the restricted adductor chamber.

The mAMEP (Fig. 3b, f) originates from the posteromedial portion of the supratemporal fossa contributed mainly by the lateral surface of the parietal, agreeing with previous interpretations^29,41,42^, The rostral boundary of the origin site is interpreted here to be just posterior to the postocular shelf. The posterolateral boundary is marked by the distinction between the fossa housing muscle origin and the head of the quadrate in *Euoplocephalus*. In *Panoplosaurus*, the posterolateral boundary is less clearly defined and a change in topological relief is interpreted as the separation between the mAMEP origin and the medial extent of the mAMEM origin. Exiting the adductor chamber, the mAMEP inserts onto the coronoid eminence of the mandible as inferred from extant sauropsids according to the EPB^41,43^. The mandibular insertion area is informed by the topological constraints imposed on the muscle path by the postocular shelf and the anterior rim of the orbit.

The mAMEM (Fig. 3b, f) originates from the posterior portion of the supratemporal fossa and inserts onto the medial surface of the surangular, posterior to the mAMEP insertion site on the coronoid eminence. In *Euoplocephalus*, the origin of mAMEM attaches onto a proportionally smaller area than it does in *Panoplosaurus* as a larger portion of the supratemporal fossa is occupied by the mAMES due to the expanded squamosal horns of the former. This difference in the size of the origin attachment area is reflected in the muscle force estimates (Table 1) as the mAMEM muscle force of *Euoplocephalus* is lower than both the original and scaled mAMEM muscle forces of *Panoplosaurus*, whereas both the forces of the other two muscles of the mAME complex are higher in *Euoplocephalus*. Since there is no clear osteological correlate defining the mandibular insertion area in both specimens, the insertion areas were chosen based on the surangular shape posterior to the coronoid eminence, the volume of the mAMEM exiting the adductor chamber and the volumetric constraints imposed by the mAMEP and mAMES.

**Table 1:** Unilateral jaw adductor muscle dimensions and force estimates of *Euoplocephalus tutus* and *Panoplosaurus mirus*.

Muscle	ML (mm)	FL (mm)	MV (mm^3^)	PCSA (mm^2^)	F_(mus)_ (N)		RMF %
*Euoplocephalus tutus*							
mAMEP	196.6	65.5	47,100.0	719.0	215.7		11.5
mAMEM	179.8	59.9	46,500.0	775.4	232.6		12.4
mAMES	189.1	63.0	64,500.0	1024.3	307.3		16.3
mPTd	127.8	42.6	36,600.0	859.5	257.9		13.7
mPTv	173.8	57.9	115,000.0	1992.7	597.8		31.7
mAMP	148.5	49.5	24,800.0	501.3	150.4		8.0
mPSTs	225.0	75.0	30,400.0	405.3	121.6		6.5
				Total	1883.2		100.0
S (mm^2^)							
867,054							
*Panoplosaurus mirus*							
mAMEP	186.7	62.2	25,500.0	410.0	122.9	173.2	8.6
mAMEM	154.5	51.5	45,000.0	874.0	262.2	368.5	18.3
mAMES	137.7	45.9	21,900.0	477.0	143.1	201.4	10.0
mPTd	110.9	37.0	16,600.0	449.0	134.7	189.3	9.4
mPTv	133.8	44.6	75,400.0	1690.0	507.2	714.9	35.5
mAMP	126.6	42.2	24,600.0	583.0	174.9	245.7	12.2
mPSTs	197.8	65.9	18,400.0	279.0	83.7	118.8	5.9
				Total	1428.7	2013.9	100.0
S (mm^2^)							
615,095							

F_(mus)_, calculated muscle force. F_(scaled)_, calculated muscle force scaled isometrically to the same surface area as *Euoplocephalus tutus*. FL, fibre length. ML, muscle length. MV, muscle volume. PCSA, physiological cross-sectional area. RMF, relative muscle force. S, total surface area of the cranium and mandibles.

The supratemporal bar, contributed by the postorbital and the squamosal, has been consistently assigned as the origin of the mAMES (Fig. 3a,e) in dinosaurs based on consistent attachment conditions found across sauropsids^36,41^. Here, the assignment of the mAMES origin sites is complicated by the closure of the supratemporal fenestra, the complete osseous fusion of the postorbital and the squamosal, and the presence of the well-developed squamosal horns in *Euoplocephalus*. In *Panoplosaurus*, the area corresponding to the supratemporal bar is much reduced compared to the typical dinosaurian condition due to the posterior displacement of the temporal region, which caused the lower temporal fenestra to be elongated and pinched posterodorsally. While the postocular shelf does provide an anterior limit to the origin site, clear osteological correlates are absent. Since the majority of the mAMES muscle seems well constrained by the descending postocular shelf and the narrow adductor chamber space lateral to the mAMEM, the origin site was selected such that the muscle could reasonably follow and fill up the path within the chamber. In *Euoplocephalus*, the expanded squamosal horns allow for in a greater room and area of attachment for the origin of the mAMES. The sub-elliptical fossa lateral to the mAMEM origin seems a reasonable site for the mAMES origin. Although the more ventrally situated, deep fossa corresponding to the squamosal horn was also considered, such an assignment would require a great expansion in the origin of the mAMEM that would result in great variation in its cross section from the origin to the insertion. Thus, the former assignment was chosen here as the more reasonable and conservative option.

On the mandible, the mAMES inserts onto the dorsal edge of the surangular in *Panoplosaurus* and the dorsal and dorsolateral surfaces of the surangular and coronoid eminence in *Euoplocephalus*. The mAMES insertion site in *Panoplosaurus* shows rugosity and connects anteriorly to a small region of depression on the dorsal surface of the coronoid eminence. This depression was initially considered for the anterior boundary of the insertion site, but the anterior rim of the orbit again imposes constraints on a more rostral attachment. In comparison, the spatial arrangement between the adductor chamber and the mandible in *Euoplocephalus* enables a more anterior attachment onto the dorsolateral surface of the coronoid eminence.

An alternative attachment site for the mAMES in herbivorous dinosaurs has been proposed more rostrally onto the labial dentary ridge lateral to the tooth row^44^. Such an attachment would facilitate palinal movements and mandibular rotation along the long axis, although we did not reconstruct this alternate attachment site due to topological constraints. In particular, we find that it was not practically possible for a muscle that needed to fan out distally to exit the adductor chamber of *Panoplosaurus* with a reasonable volume without running into the anterior rim of the orbit and the coronoid eminence. Although the *Euoplocephalus* skull has more expanded adductor chamber exits relative to the mandibles and a more rostral attachment for the mAMES is spatially supported, we find that it requires the muscle path to change from a posterodorsally directed one to a near horizontal one upon exiting the adductor chamber.

#### m. adductor mandibulae internus (mAMI) complex

The mAMI complex includes both branches of m. pseudotemporalis (mPSTs and mPSTp) and both branches of m. pterygoideus (mPTd and mPTv)^43^. The mPSTs (Fig. 3c,g) is the deepest and the most anteriorly positioned of temporal muscles and commonly originates from the anteromedial wall of the supratemporal fossa across archosaurs^36,41,43^. However, the presence of the postocular shelf prevents muscle attachment to the rostral portion of the supratemporal fossa and a hypothetical attachment onto the inner face of the postocular shelf would have interfered with the paths of the mAME muscles^29^. Furthermore, the position of the trigeminal nerve opening lies rostromedial to the postocular shelf, indicating that muscles must have originated beyond the shelf on the laterosphenoid^29,40^. Since the origin of the mPSTs has shifted onto the posterodorsal surface of the laterosphenoid in modern crocodylians, and the neuromuscular topology has been extensively researched^45^, it served as the guide here for determining the origin site for the mPSTs. In conjunction, the cranial endocasts of AMNH 5405^5^ and FWMSH93B.00026 (*Pawpawsaurus campbelli*, nodosaurid)^7,46^ were consulted for positions of the trigeminal nerve openings. For both *Panoplosaurus* and *Euoplocephalus*, the origin of the mPSTs has been reconstructed on the posterodorsal surface of the laterosphenoid, rostral to the postocular shelf and dorsal to the trigeminal nerve exit. In the absence of clear osteological correlate, the mandibular insertion sites for both specimens were inferred to be the rostromedial surface of the mandibular fossa based on the EPB^41^. The alternative possibility of inserting onto the medial surface of the coronoid eminence, as seen in lepidosaurs and neoavians^36,43^, is unlikely since the site is occupied by the mAMEP insertion and the topological constraint imposed by the pterygoid wings medially does not support both muscles attaching onto the coronoid eminence.

The mPTd (Fig. 3d,h) is the dorsal branch of the pterygoideus muscle and originates from the dorsal pterygoid and palatine^41^. In *Panoplosaurus*, we interpret an area of depression on the dorsal surface of the anteroventrally projecting pterygoid wing, marked by raised topology anteriorly and posteriorly, as the site of origin of the mPTd. Although a similar depression could not be found in *Euoplocephalus*, the dorsal surface of the pterygoid wing still seems the most reasonable place for the mPTd. To account for the more ventrally directed pterygoid wings of *Euoplocephalus* and the spatial relationship with the mPSTs, the attachment onto the dorsal pterygoid wing was placed more posteriorly in *Euoplocephalus.* The mandibular attachment site is clear for both specimens as the mPTd insertions onto medial surface of the articular and the retroarticular process^41,47^.

The mPTv (Fig. 3c,g) originates from the ventral surface of the pterygoid, posterior to the origin of the mPTd^29,41^. Here, the posteroventral portion of the pterygoid wing and the ventral surface of pterygoid with a minor portion of the quadrate process, have been interpreted as the mPTv origin sites for *Panoplosaurus* and *Euoplocephalus*, respectively. The mPTv then inserts onto the mandible along the ventral and ventrolateral surfaces of the articular and retroarticular process and thus wraps around the jaw joint and bulges out laterally.

#### m. adductor mandibulae posterior

The mAMP (Fig. 3b,f) consistently originates from the quadrate surface across sauropsids and inserts onto the medial mandibular fossa^36,41^. The spatial arrangement of the adductor chamber in relation to the mandibular fossa determines that an origin on the lateral surface of the quadrate as seen in extant bracketing taxa such as crocodylians and birds is not possible in for both studied species^41^. Instead, the rostral surface of the quadrate shows a clear tunnel-like depression likely occupied by a muscle body in life. The insertion site was assigned to the area where the depressed topology initiates, so the mAMP attaches about halfway up the rostral quadrate surface. Traditionally, the mAMP is a shorter muscle with a more expansive and ventrally placed origin attachment site on the quadrate^36^, and an alternative assignment more in line with this view is not ruled out. The insertion onto the mandibular fossa is not obviously defined by osteological markings but the way the quadrate articulates with the articular and the muscle path of the mAME complex provide constraints on what portion of the fossa mAMP likely occupied.

### Jaw muscle forces

The total muscle volume and force of the jaw adductors is greater in *Euoplocephalus* than in *Panoplosaurus* (Table 1). However, the total muscle force of *Panoplosaurus* increases 29% when scaled isometrically to the same skull surface area as *Euoplocephalus*, indicating that the former species has proportionally larger jaw muscles for its size. In both absolute and scaled sizes, the muscles of the mAME complex produce more force in *Euoplocephalus* than in *Panoplosaurus*, with the exception of the mAMEM (Table 1). When viewed as a unit, the proportional contribution by the mAME complex to the total muscle force production is comparable between *Euoplocephalus* (40.2%) and *Panoplosaurus* (36.9%), with mAMES and mAMEM being the largest contributors, respectively.

The mAMI complex contributes just over half of the adductor muscular force production in both *Panoplosaurus* (50.8%) and *Euoplocephalus* (51.9%), with mPTv being the most forceful muscle of the complex, followed by the mPTd and mPSTs. In absolute terms, the mPST and mPT of *Euoplocephalus* produce more force than those of *Panoplosaurus*, while the relationship is the opposite for mAMP. However, when scaled to the same size, the mPTv of *Panoplosaurus* becomes more forceful and both taxa have similar mPSTs muscle forces.

### Skull stress magnitudes and distribution

**Figure 4:**
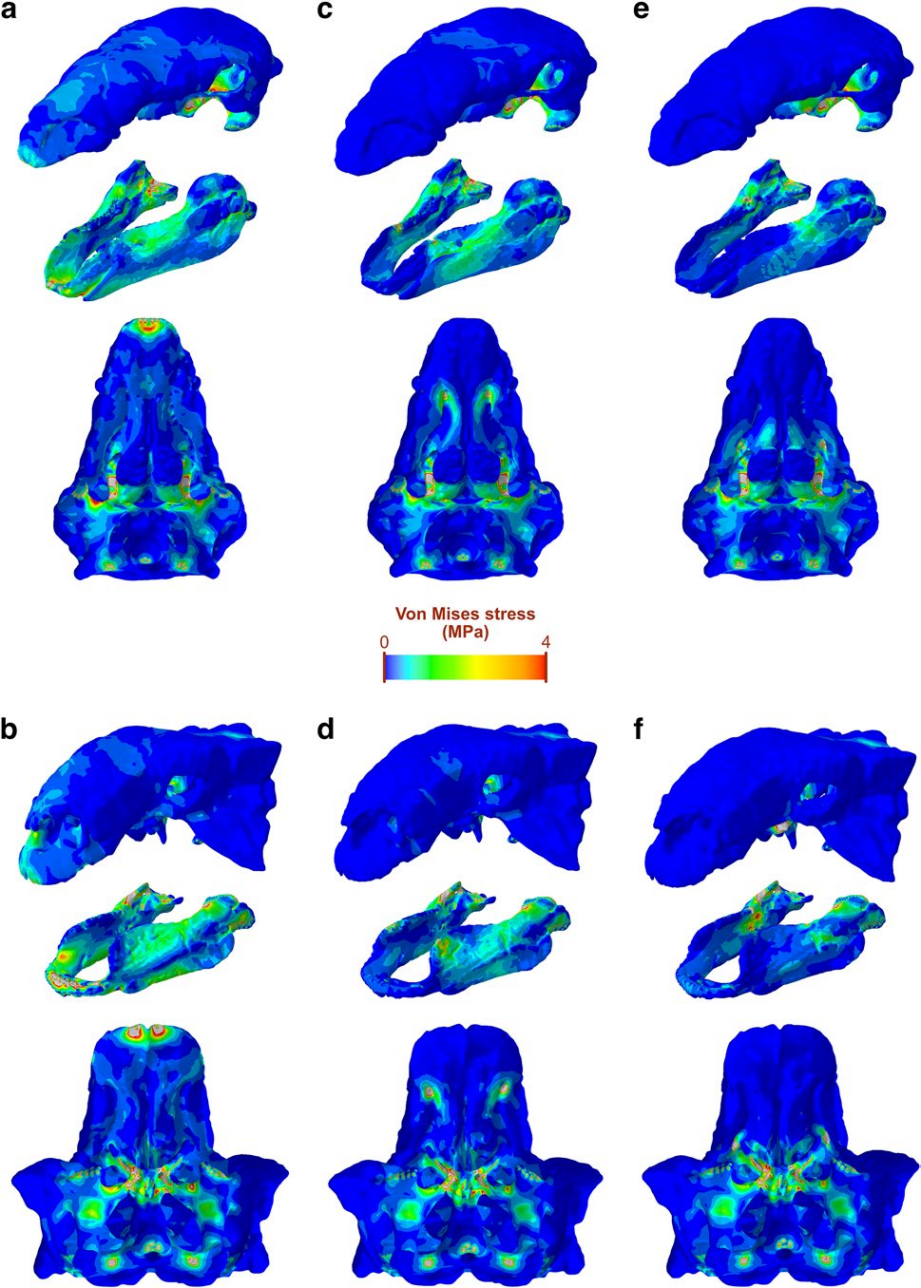
Von Mises stress distribution in the crania and mandibles of *Panoplosaurus* and *Euoplocephalus*. Biting is simulated at the tip of the muzzle (**a**, **b**), the anterior tooth position (**c**, **d**) and the posterior tooth position (**e**, **f**). Grey colour denotes areas under stress above 4 MPa.

**Figure 5:**
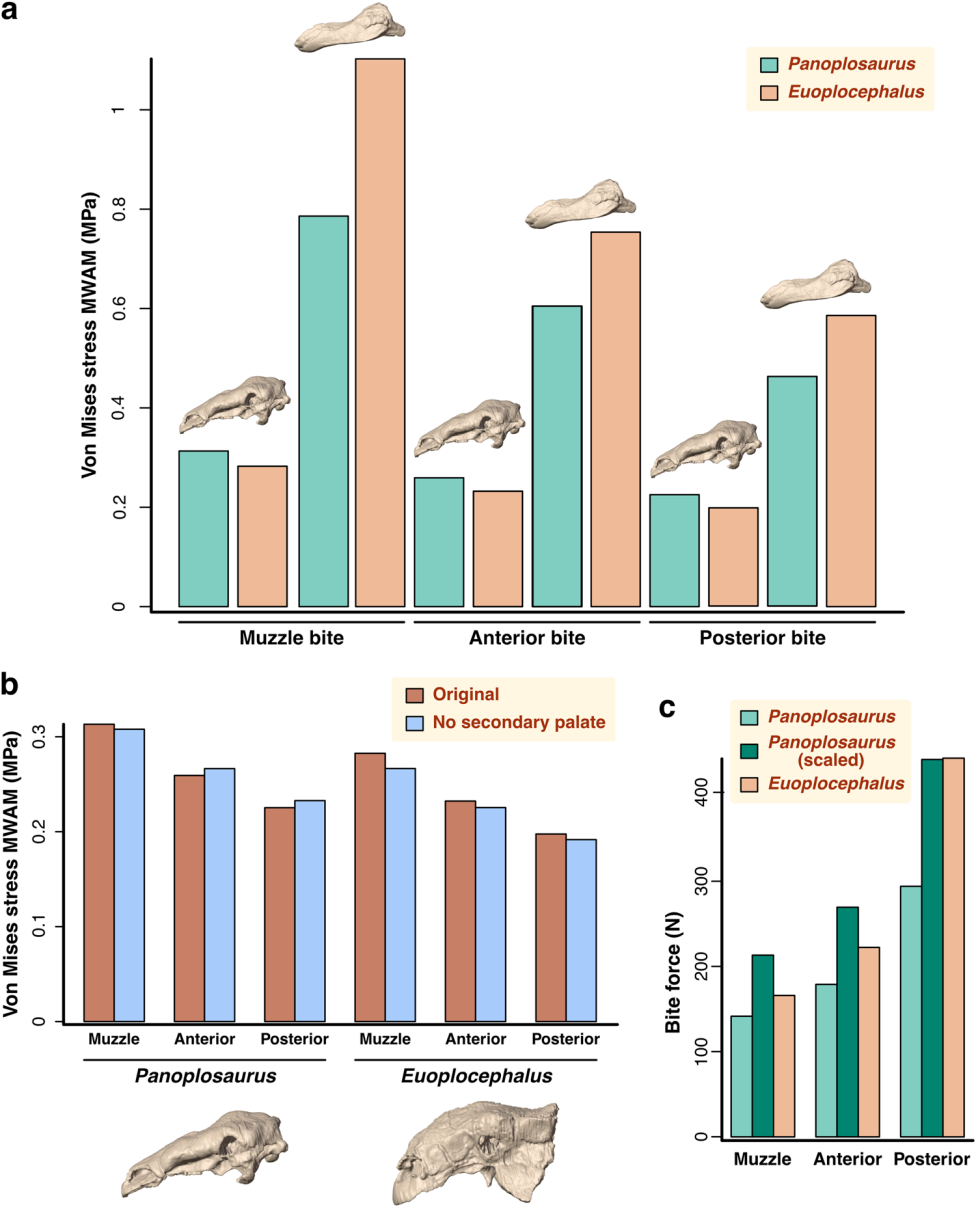
Comparative biomechanical performance of the skulls of *Panoplosaurus* and *Euoplocephalus*. (**a**) Von Mises stress mesh-weighted arithmetic mean (MWAM) values experienced by the cranium (left) and mandibles (right, as shown by 3D models above the barplot) of *Panoplosaurus* and *Euoplocephalus* under different simulated biting scenarios. (**b**) Effect of the presence or absence of secondary palate in cranial von Mises stress MWAM under different biting scenarios. (**c**) Bite force estimates obtained from the lever mechanics analysis at the three different biting positions.

We assessed von Mises stress in the skulls under three biting scenarios: tip of the jaws, anterior end of the tooth row, and posterior end of the tooth row. In all three, the stress distribution patterns in the crania of both taxa are relatively similar, with extensive areas or relatively low stress (Fig. 4). The highest levels of stress (besides where constraints were placed) are found in the pterygoid, the most consistently stressed structure of the cranium. Muscle attachment areas on the quadrate and the supratemporal fossa also experienced moderate levels of stress across the three scenarios, as well as the maxillary shelf. As the biting position moves further back along the tooth row, areas of moderate to high stress on the dorsal surface of the cranium, the ventral surface of the muzzle and along the tooth row disappear and low-stress areas extend. We note that the secondary palates of both taxa are under low to moderate stress for all biting, although higher stress is present in the vomers of *Euoplocephalus*, especially at under the muzzle and anterior biting simulations. The mesh-weighted arithmetic mean (MWAM) of von Mises stress of the crania of both *Panoplosaurus* and *Euoplocephalus* never exceed 0.4 MPa for all three simulated bites, with mean stress decreasing as the biting position moves posteriorly (Fig. 5a). In all three scenarios, the cranium of *Panoplosaurus* is under higher stress than that of *Euoplocephalus*.

In the mandibles, of the three simulated scenarios, biting at the muzzle resulted in the highest overall stress (Fig. 5a), where areas of elevated stress are spread along the mandibles (Fig. 4). Contrary to the cranium, the mandibles of *Euoplocephalus* experienced higher mean stress than mandibles of *Panoplosaurus*, and this pattern holds for all three scenarios (Fig. 5a). In addition, the mean stress on the mandibles is consistently and considerably higher than on the cranium, with the lowest mandibular mean stress (*Panoplosaurus* mandibles with a posterior tooth row bite) still exceeding 0.4 MPa. Besides areas of muscle attachment, the lateral surfaces of the dentary along the tooth row form a prominent continuous, moderately stressed band, which extends posteriorly in *Euoplocephalus* and connects with the coronoid eminence where stress exceeding 4 MPa could be found localised at the anterior portion. While the coronoid process experiences relatively high stress under all three biting scenarios for *Euoplocephalus*, this structure only experienced moderate to low stress in *Panoplosaurus*. The same also holds true for the moderately-stressed dorsolateral surface of the coronoid eminence in *Euoplocephalus.*

**Figure 6:**
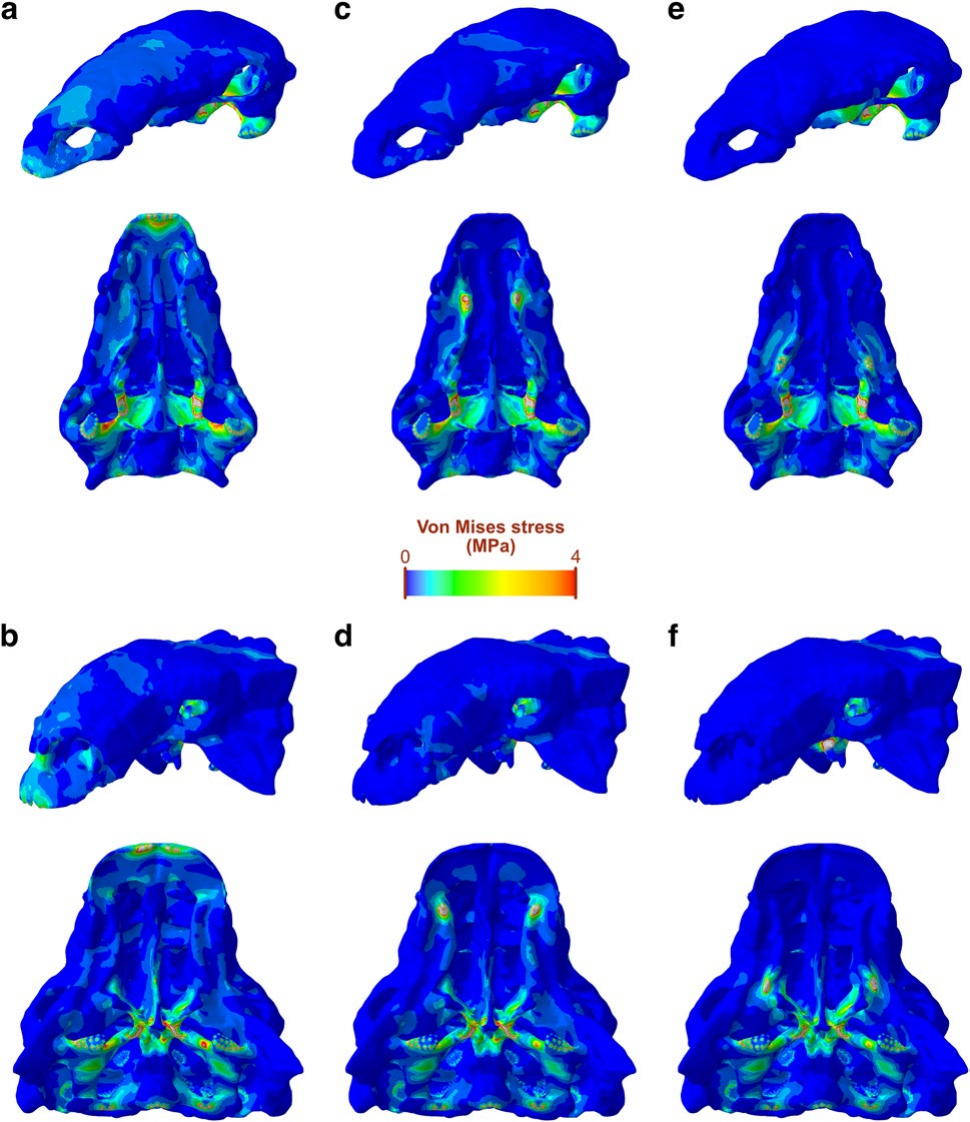
Von Mises stress distribution in the hypothetical crania without secondary palates of *Panoplosaurus* and *Euoplocephalus*. Biting is simulated at the tip of the muzzle (**a**, **b**), the anterior tooth position (**c**, **d**) and the posterior tooth position (**e**, **f**). Grey colour denotes areas under stress above 4 MPa.

The hypothetical FE models with digitally removed secondary palates show similar von Mises stress distribution patterns than the original models. Stress is similarly distributed on the dorsal surface of the crania of both species (Fig. 6). In palatal view, stress patterns do not differ greatly from the original crania, although the maxillary shelf of *Panoplosaurus* shows areas of higher stress when the secondary palate is removed, particularly under the muzzle biter scenario (Fig. 6a). In *Euoplocephalus*, the vomers are under slightly higher stress with the secondary palate removal (Fig. 6b,d,f). When the von Mises stress MWAM of the original and hypothetical models are compared, no clear pattern is observed (Fig. 5b). The removal of the secondary palate results in a slight decrease in stress in four out of six situations, excluding the anterior and posterior tooth bites of *Panoplosaurus*. However, the errors between the original and hypothetical models do not exceed 3%, except for the *Euoplocephalus* muzzle bite (6%).

### Bite force

Our bite force estimates calculated via lever mechanics (Fig. S2) show that the absolute bite force is consistently higher in *Euoplocephalus* across all tested biting positions (Fig. 5c; Table 2), and the bite force difference is the greatest when biting at the posterior tooth row position (Fig. 5c). When the bite force of *Panoplosaurus* is calculated from the isometrically scaled muscle forces to the *Euoplocephalus* skull surface area, the relationship is inverted, as *Panoplosaurus* shows greater relative bite forces at the muzzle and anterior biting positions (Fig. 5c; Table 2). Both taxa produce the same relative bite force at the posterior bite point. While not being the largest contributor to muscle force production out of the different adductor muscle groups, the mAME complex is responsible for the largest portion of bite force at all biting positions for both *Panoplosaurus* (~ 57%) and *Euoplocephalus* (62%) (Table 2). As expected, the muscle forces of the two mPT muscle branches do not translate efficiently into bite force (3–5% of muscle force) due to their short lever arms under a static bite simulation.

**Table 2:** Bite force estimates for *Euoplocephalus tutus* and *Panoplosaurus mirus* based on lever mechanics calculations.

Muscle	Sagittal angle, α (°)	Coronal angle, β (°)	F_res_ (N)	Diagonal distance (mm)	q (°)	In-lever (mm)	F(Muz) (N)	F(Ante) (N)	F(Post) (N)
*Euoplocephalus tutus* AMNH 5405									
mAMEP	38.00	14.00	172.55	59.60	68.10	55.30	33.71	45.17	90.29
mAMEM	36.00	5.30	188.56	42.70	87.30	42.65	29.55	39.59	79.13
mAMES	38.00	9.70	243.92	47.70	86.60	47.62	42.01	56.29	112.52
mPTd	34.00	37.00	229.43	17.60	17.80	5.38	3.40	4.55	9.09
mPTv	15.00	42.00	586.54	26.20	33.50	14.46	22.94	30.74	61.44
mAMP	36.00	5.00	121.87	49.10	35.50	31.40	14.07	18.85	37.68
mPSTs	20.00	20.00	115.12	81.20	38.20	50.40	20.01	26.80	53.58
						Total	165.69	221.98	443.75
Out-lever (Muz) (mm)	Out-lever (Ante) (mm)	Out-lever (Post) (mm)							
270.50	201.90	101.00							
*Panoplosaurus mirus* ROM 1205									
mAMEP	47.00	29.50	72.97	82.50	75.50	79.87	21.28	30.42	49.99
mAMEM	45.00	22.00	195.67	61.00	86.70	60.90	43.50	54.63	89.77
mAMES	49.00	21.00	99.86	56.70	78.20	55.50	20.24	25.39	41.72
mPTd	49.00	21.00	93.99	13.80	35.20	7.95	2.73	3.43	5.63
mPTv	11.00	20.00	498.92	21.10	43.30	14.47	26.36	35.33	58.06
mAMP	51.00	26.00	121.86	60.70	58.30	43.80	19.49	22.62	37.17
mPSTs	41.00	33.00	69.10	90.20	56.40	69.60	17.56	19.25	31.63
						Total	151.15	191.07	313.96
Out-lever (Muz) (mm)	Out-lever (Ante) (mm)	Out-lever (Post) (mm)							
270.50	201.90	101.00							
*Panoplosaurus mirus* ROM 1205 (scaled)									
mAMEP	47.00	29.50	102.81	82.50	75.50	79.87	29.98	42.86	70.42
mAMEM	45.00	22.00	275.04	61.00	86.70	60.90	61.15	76.79	126.18
mAMES	49.00	21.00	140.55	56.70	78.20	55.50	28.48	35.73	58.72
mPTd	49.00	21.00	132.10	13.80	35.20	7.95	3.84	4.81	7.91
mPTv	11.00	20.00	703.30	21.10	43.30	14.47	37.16	49.81	81.84
mAMP	51.00	26.00	171.18	60.70	58.30	43.80	27.37	31.77	52.21
mPSTs	41.00	33.00	98.04	90.20	56.40	69.60	24.91	27.32	44.88
						Total	212.89	269.08	442.16
Out-lever (Muz) (mm)	Out-lever (Ante) (mm)	Out-lever (Post) (mm)							
270.50	201.90	101.00							

The third panel shows scaled bite force estimates of *Panoplosaurus mirus*, calculated from isometrically scaled muscle forces to the same skull surface area as *Euoplocephalus tutus*.

α, sagittal angle of the muscle. β, coronal angle of the muscle. F_res_, resultant muscle force. d, diagonal distance from the mandibular muscle attachment sites to the jaw joint. q, the angle between the muscle line of action and d. F_(Muz)_, muscle force for bite simulated at the tip of the muzzle. F_(Ante)_, muscle force for bite simulated at the anterior tooth position. F_(Post)_, muscle force for bite simulated at the posterior tooth position. Out-lever_(Muz)_, distance from the jaw joint to the tip of the dentary. Out-lever _(Ante)_, distance from the jaw joint to first dentary tooth position. Out-lever _(Ante)_, distance from the jaw joint to last dentary tooth position.

In contrast, the adductor muscles of *Panoplosaurus* have higher mechanical advantage over their counterparts in *Euoplocephalus* in all scenarios except for posterior tooth row biting (Table 3), where the values for the mAMES, the mPTv and the mPSTs are higher in *Euoplocephalus*. As expected for jaws modelled as third-class levers, mechanical advantage increases with decreasing out-lever arms as the biting point shifts posteriorly in both species.

**Table 3:** Mechanical advantage of the jaw adductor musculature of *Euoplocephalus tutus* and *Panoplosaurus mirus*.

	*Euoplocephalus tutus* AMNH 5405			*Panoplosaurus mirus* ROM 1215		
Muscle	MA_(Muz)_	MA_(Ante)_	MA_(Post)_	MA _(Muz)_	MA _(Ante)_	MA _(Post)_
mAMEP	0.20	0.27	0.55	0.29	0.42	0.69
mAMEM	0.16	0.21	0.42	0.21	0.28	0.46
mAMES	0.18	0.24	0.47	0.19	0.25	0.42
mPTd	0.02	0.03	0.05	0.03	0.04	0.06
mPTv	0.05	0.07	0.14	0.05	0.07	0.12
mAMP	0.12	0.16	0.31	0.14	0.19	0.30
mPSTs	0.19	0.25	0.50	0.21	0.28	0.46

MA_(Muz)_, mechanical advantage calculated with the out-lever from the muzzle biting scenario. MA_(Ante)_, mechanical advantage calculated with the out-lever from the anterior tooth biting scenario. MA_(Post)_, mechanical advantage calculated with the out-lever from the posterior tooth biting scenario.

### Mechanical advantage evolution

**Figure 7:**
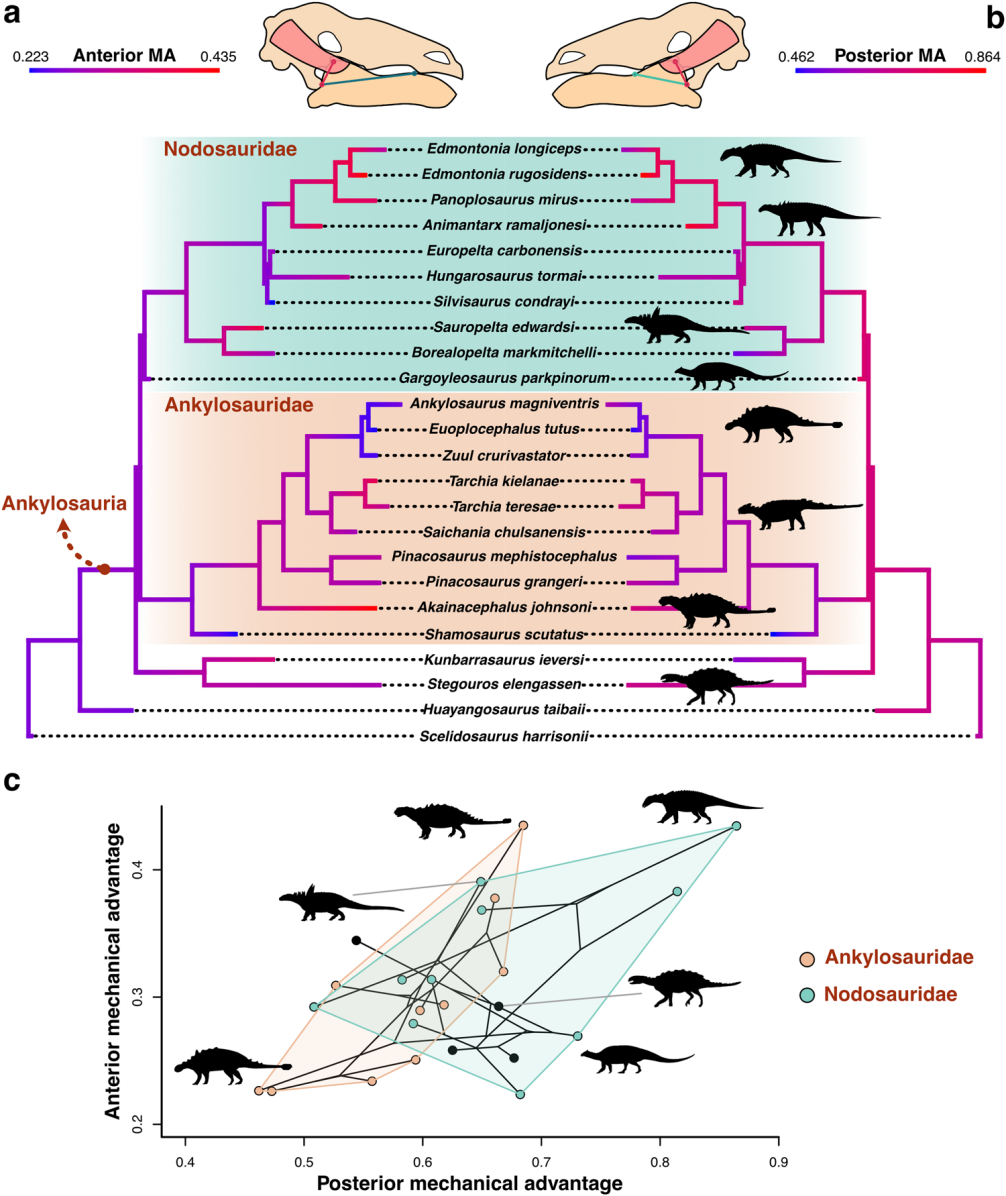
Evolution of anterior and posterior mechanical advantage of the jaw adductor muscles in Ankylosauria. Anterior (**a**) and posterior (**b**) mechanical advantage mapped onto a time-calibrated phylogeny of Thyreophora. (**c**) Phylomorphospace of anterior and posterior mechanical advantage. Silhouettes from PhyloPic (phylopic.org) and original sources.

Anterior and posterior mechanical advantage estimates of the lower jaws of 22 ankylosaur species and two outgroups within Thyreophora reveal distinct evolutionary patterns in jaw mechanics among groups. The ranges of anterior and posterior MA do not overlap, with PMA values being higher than AMA (Fig. 7a,b), as expected in mandibles. When mapped onto a time calibrated phylogeny, AMA and PMA show similar evolutionary patterns, although PMA show relatively higher values in internal branches than AMA. The two outgroups, *Scelidosaurus* and *Huayangosaurus*, have low mandibular AMA and moderate PMA, while the two early-diverging ankylosaurs (parankylosaurs) *Stegouros* and *Kunbarrasaurus* have low to moderate values of both characters. Among ankylosaurids and nodosaurids, AMA and PMA show different evolutionary patterns. While nodosaurids show generally higher PMA than ankylosaurids throughout evolution (Fig. 7b,c), opposing trends can be identified for AMA (Fig. 7a,c). The base of Nodosauridae (especially the most exclusive clade including *Silvisaurus* and *Edmontonia*) shows low PMA, which increases through to the Late Cretaceous taxa. In contrast, ankylosaurids show high PMA early in their evolution, which then sharply decreases in the latest branches. Within Ankylosauridae, the mandibles of the earliest diverging species, *Shamosaurus*, have low efficiency in terms of both AMA and PMA. Throughout the evolution of ankylosaurids, efficiency remains moderate, although the Campanian *Akainacephalus* and the Asian ankylosaurs *Tarchia* evolve jaws with relatively high AMA. In contrast, the North American ankylosaurids from the latest Cretaceous (*Euoplocephalus*, *Zuul* and *Ankylosaurus*) acquired jaws with low AMA and PMA. Regarding nodosaurids, the earliest members show moderate to highly efficient mandibles, *Silvisaurus* have mandibles with low AMA but moderate-to-high PMA, and the European taxa show moderate-to-low values of both MA metrics. Finally, *Animantarx* and the Campanian taxa *Panoplosaurus* and *Edmontonia* evolve efficient jaws with high AMA and PMA.

The phylomorphospace of AMA against PMA show that the two main ankylosaur clades overlap at the centre of the functional space (Fig. 7c). Both non-ankylosaurian thyreophorans and the parankylosaur *Stegouros* occupy a central position in terms of PMA, but towards lower values of AMA. In contract, *Kunbarrasaurus* diverges towards average values of AMA and lower PMA. The area in functional space occupied by nodosaurids is greater than that of ankylosaurids, and both clades are mostly separated along the PMA axis, with the former evolving more efficient posterior bites. Most ankylosaurids, except for *Akainacephalus* and *Tarchia*, are restricted to the lower spectrum of both AMA and PMA axes. In particular, *Shamosaurus* and the latest Cretaceous species *Euoplocephalus*, *Zuul* and *Ankylosaurus* evolved towards the lowest extremes of AMA and PMA.

## Discussion

Ornithischian dinosaurs, from a likely omnivorous ancestral condition^48–50^, evolved an outstanding diversity of craniodental adaptations for different modes of herbivory^44,47,51,52^. In contrast to the highly specialised megaherbivores like hadrosaurs and ceratopsians, ankylosaurs have been interpreted as relatively less sophisticated herbivores feeding on softer plants^40,53^. However, the current understanding of ankylosaur feeding encompasses a diversity of jaw mechanics within the clade, with some groups evolving complex biphasal mechanisms^4,24,54^. The differences in craniodental morphology, tooth wear and possible jaw function between ankylosaurids and nodosaurids suggest that these two clades specialised in feeding on different kinds of plants, the former being better suited for feeding on harder plant material^24,29^. Our study presents the first comparative 3D biomechanical assessment of ankylosaur skull function and provides further support for the divergent cranial adaptations between ankylosaurids and nodosaurids.

Our digital reconstruction of the jaw adductor muscles of *Panoplosaurus* and *Euoplocephalus* offer new insights into the biphasal jaw mechanism of ankylosaurs, the two-phase power stroke inferred in derived taxa^4^. Whereas the earliest forms had a simple jaw movement to gather and shear food materials, derived taxa in both families Nodosauridae and Ankylosauridae had evolved precise tooth to tooth occlusion, as in *Euoplocephalus* and *Panoplosaurus*. Therefore, the jaw movement consisted first of a simple orthal movement that brought the teeth into occlusion, and second a posteriorwards and slightly dorsal movement of the mandible with precise occlusion^4,54^. The overall arrangements of the reconstructed jaw adductor muscles (Fig. 3) are similar in both species in that the adductor chamber is restricted in space and well constrained by the postocular shelf. The line of action of the mAME and mAMP is notably inclined due to the orientation of the adductor chamber, and the posterior component of their generated forces likely contributed to the palinal power stroke^4,53^. Although the adductor chamber of *Euoplocephalus* is angled more vertically compared to that of *Panoplosaurus*, the resultant muscle force vector of both muscle groups clearly points posterodorsally, and thus both species could generate orthal-palinal movements of the jaw. It is, however, not clear how the adductor muscles could facilitate distinct phases of orthal and palinal movement during jaw closure. The most vertically directed muscle within the chamber is the unconventionally reconstructed mPSTs, which is not a prominent muscle in the present reconstruction. Despite the more horizontally inclined adductor chamber in *Panoplosaurus*, which may suggest that a strictly orthal stroke was not likely, tooth microwear evidence of ROM 1215 shows distinct, vertically directed scratches^4^. The orientation of the mPT branches is also consistent with the previously proposed involvement in improving tooth occlusion and palinal jaw movement by slight medial rotation of the hemimandibles^4,53,54^.

Differential traits in jaw musculature between *Euoplocephalus* and *Panoplosaurus* point at divergent jaw mechanics in ankylosaurids and nodosaurids. While the jaw adductors of *Euoplocephalus* were able to produce more force given the larger size of this specimen, when muscle forces are scaled so that the interspecific differences in size are minimised, the total jaw adductor muscle force of *Panoplosaurus* is greater (Table 1). This agrees with previous studies that examined osteological correlates of muscle attachment and suggested that nodosaurid jaw adductors were more developed than those of ankylosaurids, due to the larger adductor chamber and coronoid process of the former^4^. Similarly, the comparatively larger pterygoids of later-diverging nodosaurids like *Panoplosaurus* were interpreted as evidence for more developed mPT^4^. Our 3D reconstruction shows that, in relative terms, *Panoplosaurus* had a significantly larger mPTv and the combined force of its mPT complex was greater than that of *Euoplocephalus*. The more powerful jaw musculature of *Panoplosaurus* appears to be one of the traits linked to the harder and mechanically demanding diet of nodosaurids^4,24,29^.

Our FE models show that the cranium and mandibles of both species are only slightly stressed by simulated feeding-related forces acting upon them. The low stress levels on the dorsal surface of the crania of both species (Fig. 4) are not only related to the relatively modest jaw musculature, but also to the extensive ornamentation and ossification of the skull, an apomorphic trait of ankylosaurs^55^. The mechanically resistant configuration of the ankylosaur cranium might be interpreted as part of the overall body armour to defend against large predators or to use in intraspecific combat. Under all simulations, *Euoplocephalus* shows lower stress in the cranium than *Panoplosaurus*, consistent with the more elaborate and ossified skull of ankylosaurids. While our analyses simulate forces generated by the jaw adductors and not external loads, the results suggest that the cranium of *Euoplocephalus* was better suited for defensive behaviours. Given the presence of tail clubs in ankylosaurids, which were mechanically suited for impacts^16,56^, this suggests that more structurally resistant crania evolved in combination with their possibly more mechanically demanding intraspecific combat mode when compared to nodosaurids. Apart from the extensive low-stress areas on the dorsal surface, high stress concentrates on the ventral side of the cranium, especially in the pterygoids. An interesting result is the relatively high stress present in the vomers of *Euoplocephalus* compared to *Panoplosaurus*, which increases further with the removal of the secondary palate. Fused vomers with expanded ventral processes have been reported in *Panoplosaurus* and *Edmontonia*^57,58^, a feature that has been associated with complex oral processing or stress dissipation under high bite forces^29,58^. Our findings support the idea that the fused vomers of *Panoplosaurus* were somehow involved in resisting feeding-related forces, at least in relative terms compared to the ankylosaurid *Euoplocephalus*.

In contrast with the cranium, the mandible of *Euoplocephalus* is consistently under higher stress than that of *Panoplosaurus*. The main function of the vertebrate mandible is to transmit forces generated by the jaw muscles, and thus its relationship to feeding is considered tighter than that of the cranium, which is involved in many other functions (e.g., protection of the brain and sensory organs, configuration of the upper air pathways). Our comparative analyses reveal that while the lower cranial stress of *Euoplocephalus* might be resulting from its more heavily ornamented cranium with highly defensive function, the skull of *Panoplosaurus* was better suited for withstanding feeding related forces at the mandible. The mandible of *Panoplosaurus* is more robust than that of *Euoplocephalus*, which in turn shows a region of relatively high stress at the coronoid eminence lacking in the former. This differentiates the two main ankylosaur clades, as nodosaurids have more developed coronoid processes than ankylosaurids. Our results show that the more developed coronoid process of *Panoplosaurus*, and by extension nodosaurids, improved feeding efficiency by increasing the mechanical advantage of the muscles inserting on this area (mAME and mAMP, Table 3) as well as alleviating elevated stresses resulting from feeding.

An osseous secondary palate has convergently evolved in different groups of amniotes, such as mammals, turtles and crocodilians. In these groups, the evolution of this structure has been linked to feeding, since it is involved in resisting loads and reinforcing the skull^20,21^. A secondary palate has also evolved in different lineages of dinosaurs, such as spinosaurid theropods^59^ and ankylosaurs^17^. Biomechanical modelling of this structure in spinosaurs supports its mechanical role in this clade too, conferring resistance to the skull against bending^22^. Our study presents the first biomechanical assessment of the role of the secondary palate in ankylosaurs. This structure is simpler in nodosaurids, composed of only of an anterodorsal palate made of the vomers, premaxillae and maxillae, while ankylosaurids also have a second part, the posteroventral secondary palate, composed of the palatines and pterygoids^1,17,18^. Comparison of mean von Mises stress between original and hypothetical FE models of *Panoplosaurus* and *Euoplocephalus* do not show a clear trend in stress reduction or increase (Figs. 5b, 6), suggesting that the overall contribution of the secondary palate to reinforce the skull against feeding loads is minimal, for both nodosaurid and akylosaurid palate morphotypes. This indicates that in the case of dinosaurs, the secondary palate plays an important mechanical role in longirostrine forms^22^, but not in the brevirostrine skulls of ankylosaurs. In contrast, the evolution of this structure in ankylosaurs might be linked to the acquisition of complex nasal passages, which represented an adaptation for efficient thermoregulation^60^. Thus, the secondary palate of ankylosaurs appears to serve strictly as a separation between the respiratory airways and the mouth.

Previous studies suggested that nodosaurids might have possessed more efficient jaw mechanisms than ankylosaurids based on relative bite forces (RBF), equivalent to the mechanical advantage^29,61^. Here, we calculated MA and RBF beyond the mAME complex, unlike in previous studies, and our results largely corroborate previous findings. Overall, the consistently higher mechanical advantage of the muscles of *Panoplosaurus* (Table 3) suggests that its mandibular musculoskeletal system was more efficient at converting muscle forces into bite forces than *Euoplocephalus*. However, we also found that *Euoplocephalus* was able to deliver greater absolute bite forces despite being less efficient. This is likely the result of the larger size of the *Euoplocephalus* specimen, since the bite force of *Panoplosaurus* is greater at the muzzle and anterior bite points when the effect of size is removed by isometrically scaling the muscle forces. This suggests that in relative terms *Panoplosaurus* had a more efficient feeding apparatus which was also capable of producing more powerful bites than *Euoplocephalus*. These results support the view that nodosaurids in general may have been better adapted for consuming tougher food than ankylosaurids^24,29^, or at least that *Panoplosaurus* was more efficient at doing so than the sympatric *Euoplocephalus*, further confirming the hypothesis of niche partitioning between the two clades.

Herbivorous dinosaurs evolved a diverse array of jaw mechanics associated with different feeding modes. Our models reveal that late diverging ankylosaurs showed a constrained range of bite forces (*Panoplosaurus*, 141–294 N; *Euoplocephalus*, 166–444 N) within the average of dinosaurian herbivores. These bite force magnitudes are comparable to those of *Stegosaurus stenops* (231–410 N), another herbivorous thyreophoran of large body size^36^, and above the range of bite forces estimated for most herbivorous theropods (i.e., oviraptorosaurs, ornithomimosaurs and therizinosaurs)^62–64^ and the early diverging sauropodomorph *Plateosaurus*^36,65^. When compared to sauropods, the bite forces of *Panoplosaurus* and *Euoplocephalus* are similar to those of the gracile-skulled *Diplodocus* (235–324 N) but noticeably lower than those of the robust *Camarasaurus* (982–1859 N)^66^. Bite force data based on volumetric muscle reconstructions is missing for hadrosaurs and ceratopsians, although estimates from alternative methods are much higher (hadrosaurs, 317–775 N; ceratopsians, 437–1131 N)^67^ than our estimates for ankylosaurs. This diversity of bite performance estimates suggests that herbivorous dinosaurs specialised in different ways to a plant-based diet. Late Cretaceous ankylosaurs had bite forces similar to those of other large obligate herbivorous dinosaurs such as stegosaurs and diplodocids which likely fed on softer plants and lacked adaptations for extensive oral processing^36,65^. The higher bite forces of ankylosaurs relative to herbivorous theropods are mostly due to overall size in the case of oviraptorosaurs^64^, and to the gracile skulls with small jaw adductors of ornithomimosaurs and therizinosaurs^62,63^. In contrast, ankylosaurs had weak bites compared to megaherbivorous dinosaurs that used extensive oral processing on harder plant material such as some sauropods^66^, and most importantly, hadrosaurs and ceratopsians^67^. The clearly distinct bite performance of *Panoplosaurus* and *Euoplocephalus* relative to hadrosaurs and ceratopsians recovered in this study provides further evidence for niche partitioning between these three sympatric ornithischian clades in Late Cretaceous North America^29^. Ankylosaurs evolved a different functional approach to herbivory compared to other sympatric ornithischians, with weaker bite forces suited for softer plants.

The evolution of mechanical advantage in ankylosaurs reveals divergent trends in jaw mechanics between ankylosaurids and nodosaurids (Fig. 7). The earliest diverging ankylosaurs show moderate-to-low values of AMA and PMA, and these taxa are thought to have lacked tooth occlusion and have a simple orthal jaw mechanism^4^. Among nodosaurids and ankylosaurids, a number of species converged on average values of mechanical efficiency, while the late-diverging lineages of each clade specialised in opposing modes of jaw mechanics. The ankylosaurids of the latest Cretaceous, such as *Euoplocephalus*, *Zuul* and *Ankylosaurus*, evolved jaws with low mechanical advantage, coupled with tooth occlusion and a complex biphasal jaw mechanism^4,24,54^. While this complex tooth occlusion and jaw movement evolved convergently in the Late Cretaceous nodosaurids *Panoplosaurus* and *Edmontonia*^4,24^, these species also evolved mandibles with high mechanical advantage. Thus, despite the convergent acquisition of complex oral processing by the latest members of the two main ankylosaur clades, nodosaurids also evolved efficient jaws capable of producing higher relative bite forces than ankylosaurids, suggesting divergent strategies in jaw mechanics and dietary partitioning, as previously suggested from different lines of evidence^4,24,28,29^. Our results also suggest that the evolution of AMA and PMA are decoupled in ankylosaurids and nodosaurids. Nodosaurids maintained generally higher PMA relative to ankylosaurids throughout evolution, but AMA tended to decrease in Ankylosauridae and increase in Nodosauridae. This is reflected in the early diverging ankylosaurids *Akainacephalus* and *Tarchia*, which show high mandibular AMA compared to other species. Asian ankylosaurids like *Tarchia* are characterised by the lack of tooth occlusion and are thought to have processed food by simple orthal pulping^4^. The presence of highly efficient anterior bites in these species suggests a mechanism for countering the simple oral processing or, most likely, an adaptation for efficient food prehension^68^ of xeric plants in their arid habitats, possibly relating this to the interpretation of *Tarchia* as selective feeders^69^. The Early Cretaceous nodosaurid *Sauropelta* also shows elevated AMA, although this species had full tooth occlusion and a complex jaw mechanism like its Late Cretaceous relatives *Panoplosaurus* and *Edmontonia*^4^. AMA decreases in later branches including species like *Hungarosaurus* and *Silvisaurus* before increasing again in the clade including *Animantarx* and the Campanian taxa. This suggests that the acquisition of complex jaw mechanics coupled with efficient anterior leverage evolved convergently among different nodosaurid lineages, possibly associated with an increase in plant prehension efficiency.

In conclusion, our study presents the first computational biomechanical assessment of ankylosaur skull function, revealing notable differences between the nodosaurid *Panoplosaurus* and the ankylosaurid *Euoplocephalus*. *Panoplosaurus* had proportionally larger jaw adductors and efficient mandible, capable of better resisting feeding-related loads and producing relatively higher bite forces. On the other hand, *Euoplocephalus* shows a remarkably reinforced cranium, suggestive of a highly efficient defensive function. In both taxa, the secondary palates are not involved in reinforcing the skull against feeding loads. Finally, evolutionary trends in mandibular mechanical advantage reveal that late-diverging ankylosaurids evolved mandibles with low MA jaws while nodosaurids evolved highly efficient jaws.

## Material and methods

### Institutional abbreviations

AMNH, American Museum of Natural History, New York, USA; FWMSH, Fort Worth Museum of Science and History, Fort Worth, USA; ROM, Royal Ontario Museum, Toronto, Canada.

### Anatomical abbreviations

mAMES, m. adductor mandibulae externus superficialis; mAMEM, m. adductor mandibulae externus medialis; mAMEP, m. adductor mandibulae externus profundus; mAMI, m. adductor mandibulae internus; mAMP, m. adductor mandibulae posterior; mLAO, m. levator anguli oris; mPSTs, m. pseudotemporalis superficialis; mPSTp, m. pseudotemporalis profundus; mPTd, m. pterygoideus dorsalis; mPTv, m. pterygoideus ventralis.

### 3D model creation

Three-dimensional digital models of the skulls and mandibles of the ankylosaurid *Euoplocephalus tutus* (AMNH 5405) and the nodosaurid *Panoplosaurus mirus* (ROM 1215) were constructed from previously published CT scan data^70^ via the WitmerLab webpage (https://people.ohio.edu/witmerl/lab.htm). Permission to use the CT data was obtained from the author L.M. Witmer (Ohio University Heritage College of Osteopathic Medicine). CT data of AMNH 5405 include scans of the complete skull, the right mandible and the predentary. CT data of ROM 1215 include scans of the complete skull and the left mandible. The fossil materials of both specimens were collected from the Dinosaur Park Formation (Upper Cretaceous, Campanian) of Alberta, Canada and CT scanning parameters can be found in the original publication^70^. CT scans (DICOM files) were imported into Avizo 3D 2021.1 (FEI Visualization Sciences Group; Thermo Fisher Scientific) to create 3D digital models of the skulls. This was achieved by manual segmentation of the fossilized bone and iterative interpolation between slices in the Avizo Segmentation Editor, carefully removing sediment infills from the segmentation. A first digital restoration step was conducted in Avizo using the tools in the Segmentation Editor. Elements that suffered taphonomic damage were repaired using techniques laid out in Lautenschlager^71^, such as interpolation to remove cracks in the fossils. 2D surface models were exported from the segmentation and subsequently imported into Blender (v.2.93; Blender Foundation; www.blender.org) to create retrodeformed, articulated skull-mandible units of both specimens.

Retrodeformation procedures were performed on both skulls via the Lattice Modifier in Blender (Fig. 1). Despite being reasonably complete, both skulls were deformed during burial. In particular, the *Euoplocephalus* skull suffered a considerable degree of plastic deformation laterally on the left half but was minimally deformed dorsoventrally^72^. Although this skull was retrodeformed in a previous study utilizing the orbits as strain ellipses^72^, here a simpler approach was taken by using the less deformed right half as well as ventral elements as references (pterygoid and vomer) to restore bilateral symmetry. The same approach was taken for the *Panoplosaurus* skull, which required only minor adjustments. The hemimandibles were duplicated, mirrored, and joined to meet the quadrates in the undistorted skulls, producing the models to be used in the functional analyses (Fig. 1).

Hypothetical skull morphologies of *Euoplocephalus* and *Panoplosaurus* lacking the secondary palate were created in order to test the mechanical role of this structure in reinforcing the skull against feeding-related loads. The corresponding horizontal portions of the premaxillae and maxillae that form the anterodorsal secondary palate in both taxa, and the contributions of the palatines and pterygoids to the posteroventral secondary palate of *Euoplocephalus* were segmented out in Avizo 3D 2021.1. The models were later cleaned and smoothed in Blender to remove surface artefacts and generate the final hypothetical skull morphologies without secondary palates (Fig. 2).

### Digital muscle reconstruction

3D reconstructions of the jaw adductor musculature of both specimens were carried out following an established protocol^62^, widely used in palaeo-biomechanics^63,64,66,73,74^. We followed the initial step of identifying muscle attachment sites on the models based on osteological correlates as well as considerations of topological, neurovascular and homological criteria (Fig. S1). The subsequent step of creating straight-line connections between origin and insertion sites via simplified cylinders was modified by using ‘MyoGenerator’, a Blender add-on for volumetric muscle construction within Blender^75^. Instead of simplified cylinders, ‘MyoGenerator’ creates adjustable muscle curves by making non-uniform rational B-splines (NURBS) paths connecting the centroids of the origin and insertion sites, which has the advantage of outputting more detailed volumes and muscle lengths than traditional methods^75^. The final step of iteratively “fleshing out” the volumetric muscles was done by adjusting the muscle curves with the transform and sculpting tools in Blender.

Previously proposed reconstructions of jaw adductor muscles for ankylosaurs^4,29,40–42^ and other herbivorous dinosaurs^36,47,66^ served as guides for identifying muscle attachment sites. Where different reconstructions disagree, topological constraints and established homological inferences for non-avian dinosaurs based on the Extant Phylogenetic Bracket approach (EPB)^41,76^ were preferentially used to inform decisions.

Seven branches of jaw adductor muscles were chosen for reconstruction for both specimens. The m. levator anguli oris (mLAO) is not reconstructed here despite its inclusion in previous reconstructions^40,42^. The absence of this muscle among extant crocodilians and birds, two groups which phylogenetically bracket dinosaurs, does not support its reconstruction in our specimens based on the weak inference level (level III’) according to the EPB^29,36^. The m. pseudotemporalis profundus (mPSTp) is also not included in our reconstructions as EPB indicates that this muscle likely originates from the surface of the epipterygoid^41^. Despite the presence of the epipterygoid in Ankylosauria^1,8,47^, this bone is not preserved in either specimen under study here and is thought to have been lost in other derived ornithischian clades (Hadrosauridae and Ceratopsidae), resulting in the loss of mPSTp or its replacement with a ligament^41,77^. Therefore, the mPSTp in the two study species, if present, likely did not play a major role in force production or cranial stress distribution during jaw closure.

### Muscle force estimation

Estimates of muscle force for all muscles were calculated (Eq. 1) following a variation of the “dry skull” method^78^, where muscle cross-sectional area (CSA) is multiplied by the isometric stress value (*σ*) of 0.3 N mm^−2^.

1 $${\mathrm{F}}_{\mathrm{mus}}=\mathrm{CSA}\times\upsigma$$

The cross-sectional area is calculated as the muscle volume (MV) divided by muscle fibre length (FL)^38,62,79^, where FL is approximated as a third of the muscle length (ML/3). Values of MV and ML were obtained through the ‘MyoGenerator’ add-on, which outputs the muscle volume and muscle length among other positional information. The add-on calculates ML by summing the lengths of the edges constituting the muscle curves. Muscle force estimates of *Panoplosaurus* scaled to the same surface area to total muscle force ratio as *Euoplocephalus* were calculated to allow for appropriate comparisons of functional performances between the skulls of the two species without the effect of size^80^.

### Finite element analysis

The surface models of the crania and mandibles were remeshed and cleaned with the Remesh Modifier and the mesh cleaning tools in Blender. After this, the surface models were imported into Hypermesh 2021.2 (Altair Engineering; https://altairhyperworks.co.uk/) for 3D meshing. The crania and mandibles were meshed into models of approximately 4 million tetrahedral elements (Table S1). All FE models were assigned the material properties of alligator mandible: Young’s modulus (*E*) = 15 GPa, Poisson’s ratio (υ) = 0.29^38,81^ and treated as isotropic and homogeneous. Although assuming material isotropy will lead to less realistic absolute value outputs, this approach was taken because anisotropic properties cannot be reliably determined for fossil materials and studies have shown that such assumptions do not prevent meaningful comparisons of relative stress and strain distributions across models^36,82^.

All meshed models were imported from Hypermesh into Abaqus (v6.14; Simulia) where nodal constraints, boundary conditions and loads (muscle force estimates) were applied (Table S2). FE models simulated static, bilateral biting at three biting positions: the tip of the jaws (i.e., anterior tip of the muzzle), anterior end of the tooth row (i.e., anteriormost tooth position) and the posterior end of the tooth row (i.e., posteriormost tooth position). For each of the two crania, all degrees of freedom were constrained at the occipital condyle, the paraoccipital processes and the quadrate condyles. For each set of mandibles, all degrees of freedom were constrained at the glenoid fossa. To simulate the three biting scenarios, fully constrained nodes were placed at the first and last maxillary and dentary tooth positions, as well as at the tips of the edentulous muzzles.

All models were loaded with muscle forces (Table 1) by applying concentrated forces of the estimated values to areas of muscle attachment denoted by nodes (30–90 nodes) and with force vectors following the directions of the muscle paths. For the cranium and mandibles of *Panoplosaurus*, scaled forces were applied instead, which were obtained by scaling to the same total muscle force to skull surface area as *Euoplocephalus* (Table S2). Analyses under the same settings were performed for the hypothetical crania without secondary palates of both taxa.

Contour plots of von Mises stress, a metric that predicts failure under ductile fracture^31,83^, were produced to qualitatively assess and compare the stress (tensile and compressive) experienced by the crania and mandibles under the different simulated biting scenarios. To quantitatively compare stress responses, mean von Mises stress values were computed for each FE model, using the Mesh-Weighted Arithmetic Mean (MWAM), which accounts for mesh heterogeneity and minimises the effect of artefactually high values^84^. These calculations were run in RStudio v. 2022.7.1.554 using an R script modified from Ballell and Ferrón^85^.

### Bite force and mechanical advantage calculation

Bite force estimates at the three biting positions were obtained for *Euoplocephalus* and *Panoplosaurus* by treating the jaws as third-class, two-dimensional lever systems^62^. For *Panoplosaurus*, calculations were conducted using both the original and scaled muscle forces, the former being obtained by isometrically scaling to the same skull surface area as *Euoplocephalus*. The resultant muscle forces (F_res_) were calculated from the angles of muscle insertion measured in the sagittal (α) and coronal (β) planes (Eq. 2). The bite force estimates (F_bite_) were derived from the relationships between forces and lever arms in lever systems (Fig. S2, Eq. 3). Here, the out-lever (L_out_) is the distance from the jaw joint to the biting point and the in-lever (L_in_) is the perpendicular distance from the jaw joint to the line of action of the muscle. Lengths of the in-levers were estimated using Eq. (4), modified from Ostrom^86,87^, where d is the diagonal distance from the jaw joint to the mandibular insertion site (maximum length for input lever) and q is the angle between the muscle line of action and d. The angle q here is equivalent to the combined angle of θ and δ from previous studies that have applied this method to estimate bite force in ornithischian dinosaurs^29,61,86,88^, whose maximum angle is π/2. For muscles with q measurements larger than π/2, the supplementary angle of q was used instead^87^. Differing from previous works that restricted bite force calculations to mAME^29,61,86,88^, the present study presents calculations for all seven reconstructed muscles.

2 $${\mathrm{F}}_{res}={\mathrm{F}}_{mus}\times \mathrm{cos\alpha }\times \mathrm{cos\beta }$$

3 $${\mathrm{F}}_{\mathrm{bite}}\times {\mathrm{L}}_{\mathrm{out}}={\mathrm{F}}_{\mathrm{res}}\times {\mathrm{L}}_{\mathrm{in}}$$

4 $${\mathrm{L}}_{\mathrm{in}}=\mathrm{sin\theta }\times \mathrm{d}$$

In addition to bite force estimates, the mechanical advantage (MA) of each muscle at each biting position was also calculated with Eq. (5) as a measure of mechanical efficiency in force transfer of the jaw and associated muscles. These values allowed comparisons with relative bite force (RBF) estimates from previous studies^29,61^.

5 $$\mathrm{MA}=\frac{{\mathrm{L}}_{\mathrm{in}}}{{\mathrm{L}}_{\mathrm{out}}}$$

### Mechanical advantage evolution

The mandibular mechanical advantage of 22 species of ankylosaurs and two outgroups (the stegosaur *Huayangosaurus* and the early-diverging thyreophoran *Scelidosaurus*) were measured from images of skulls with preserved lower jaws (Table S3). The sample includes two early-diverging ankylosaurs, 10 ankylosaurids and 10 nodosaurids. Mechanical advantage was calculated using Eq. (5), and measurements of in- and out-levers in 2D were taken in ImageJ 1.53 k (National Institutes of Health, USA). The out-lever was measured from the jaw joint to the biting points, at both the anterior and posterior ends of the tooth row, to obtain estimates of anterior and posterior MA. The in-lever was measured from the jaw joint to the centre of the insertion area of the mAME complex, represented by the dorsal outline of the coronoid process in medial/lateral view. This simplified measurement has been commonly used as an approximation of in-levers in macroevolutionary analyses of mechanical advantage^38,89–91^. Lever arm measurements and MA values are provided in Table S4.

A simplified phylogeny of Ankylosauria was time-calibrated to reconstruct the evolution of mechanical advantage. The phylogeny presented in Soto-Acuña et al.^15^ was pruned to include the 24 taxa in our dataset. Species first and last appearance data were derived from the formations and stages of provenance and the chronostratigraphic ages from Gradstein et al.^92^. The phylogenetic tree was time-scaled based on the species age ranges using the “equal” method in the timePaleoPhy function of the “paleotree” R package^93^. The calibrated phylogeny was plotted against the geologic time scale (Fig. S3) using the geoscalePhylo function in the “strap” package^94^.

The evolution of jaw mechanics in Ankylosauria was investigated using phylogenetic comparative methods. Anterior and posterior mechanical advantage of the jaws were mapped onto the time-calibrated phylogeny using the contMap function of the “phytools” package in R^95^, based on maximum likelihood ancestral state estimation of internal nodes (Fig. 7a,b). In addition, a phylomorphospace of anterior and posterior MA was generated to represent the similarities in jaw mechanics among taxa and their phylogenetic relationships (Figs. 7c, S3). This was achieved using the phylomorphospace function in “phytools".

## Supplementary Information

Supplementary Information.

## Data Availability

The original CT datasets are available at https://people.ohio.edu/witmerl/lab.htm. Models created in this research are available on Figshare at https://doi.org/10.6084/m9.figshare.23599374.v1.
