# Author Correction: Divergent strategies in cranial biomechanics and feeding ecology of the ankylosaurian dinosaurs

**DOI:** 10.1038/s41598-023-46872-9

**Published:** 2023-11-09

**Authors:** Antonio Ballell, Bohao Mai, Michael J. Benton

**Affiliations:** Bristol Palaeobiology Group, School of Earth Sciences, Life Sciences Building, University of Bristol, Tyndall Avenue, Bristol, BS8 1TQ UK

Correction to: *Scientific Reports* https://doi.org/10.1038/s41598-023-45444-1, published online 25 October 2023

The original version of this Article contained an error in Figure 3, where panel **d** was a duplication of panel **c**. The original Fig. 3 and accompanying legend appear below.

**Figure 3:**
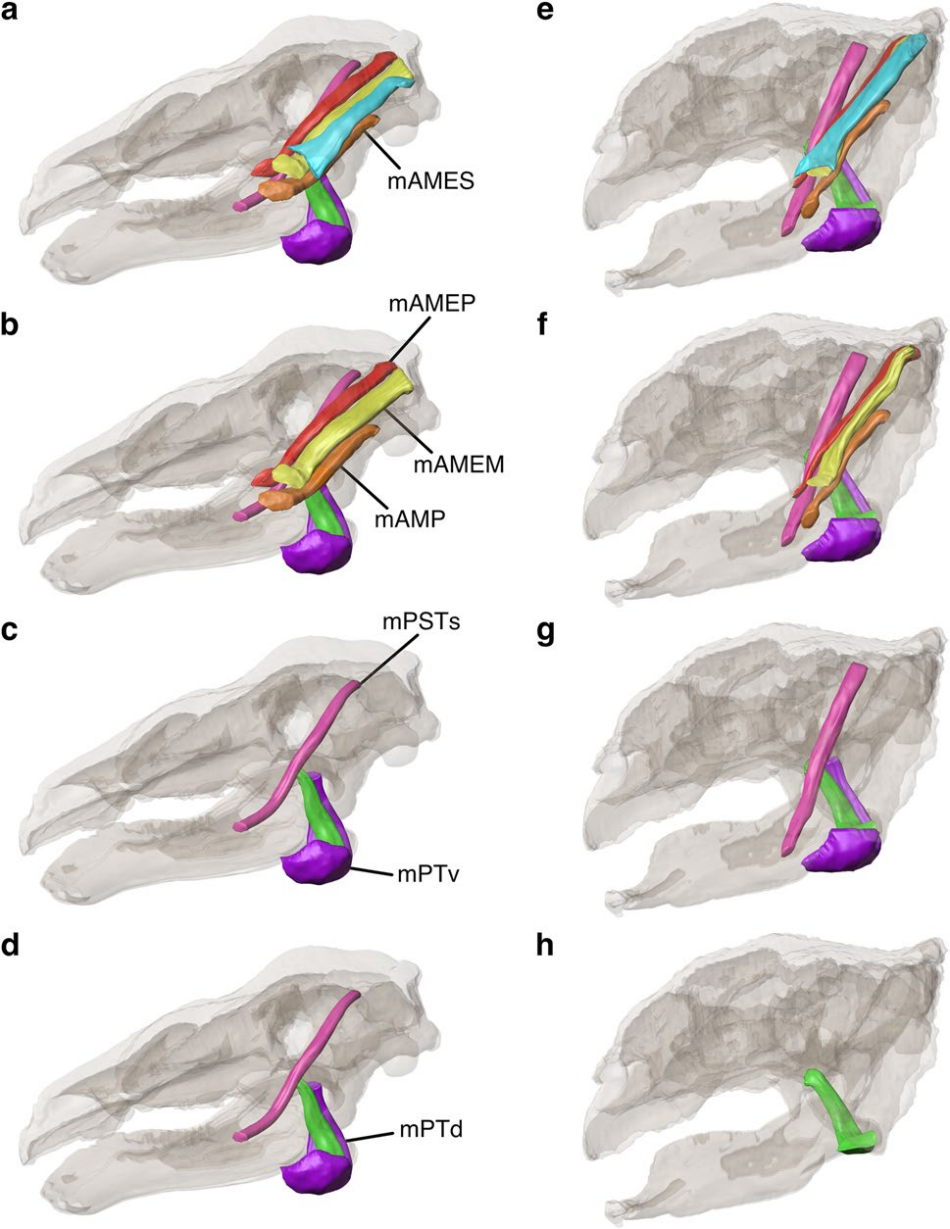
Digitally reconstructed jaw adductor muscles of *Panoplosaurus mirus* (**a**–**d**) and *Euoplocephalus tutus* (**e**–**h**) with skulls shown in left lateral view. (**a**,**e**) all reconstructed muscles. (**b**,**f**) mAMES removed. (**c**,**g**) mAMEM, mAMEP and mAMP removed. (**d**,**h**) mPSTs and mPTv removed.

The original Article has been corrected.

