# Supplementary material for: Divergent strategies in cranial biomechanics and feeding ecology of the ankylosaurian dinosaurs
Source: Sci Rep. 2023 Oct 25;13:18242. doi: 10.1038/s41598-023-45444-1 (PMC10600113; doi:10.1038/s41598-023-45444-1)
Supplement: Supplementary file 1 — Supplementary Information. [file 41598_2023_45444_MOESM1_ESM.pdf]

Supplementary Information

Divergent strategies in cranial biomechanics and feeding ecology of the ankylosaurian dinosaurs

Antonio Ballell[1]*, Bohao Mai[1] and Michael J. Benton[1]*

[1]Bristol Palaeobiology Group, School of Earth Sciences, University of Bristol, Life Sciences Building, Tyndall Avenue, Bristol, BS8 1TQ, UK

*antonio.ballell@bristol.ac.uk; mike.benton@bristol.ac.uk

This file includes:

Supplementary Figures 1–4

Supplementary Tables 1–4

Supplementary References

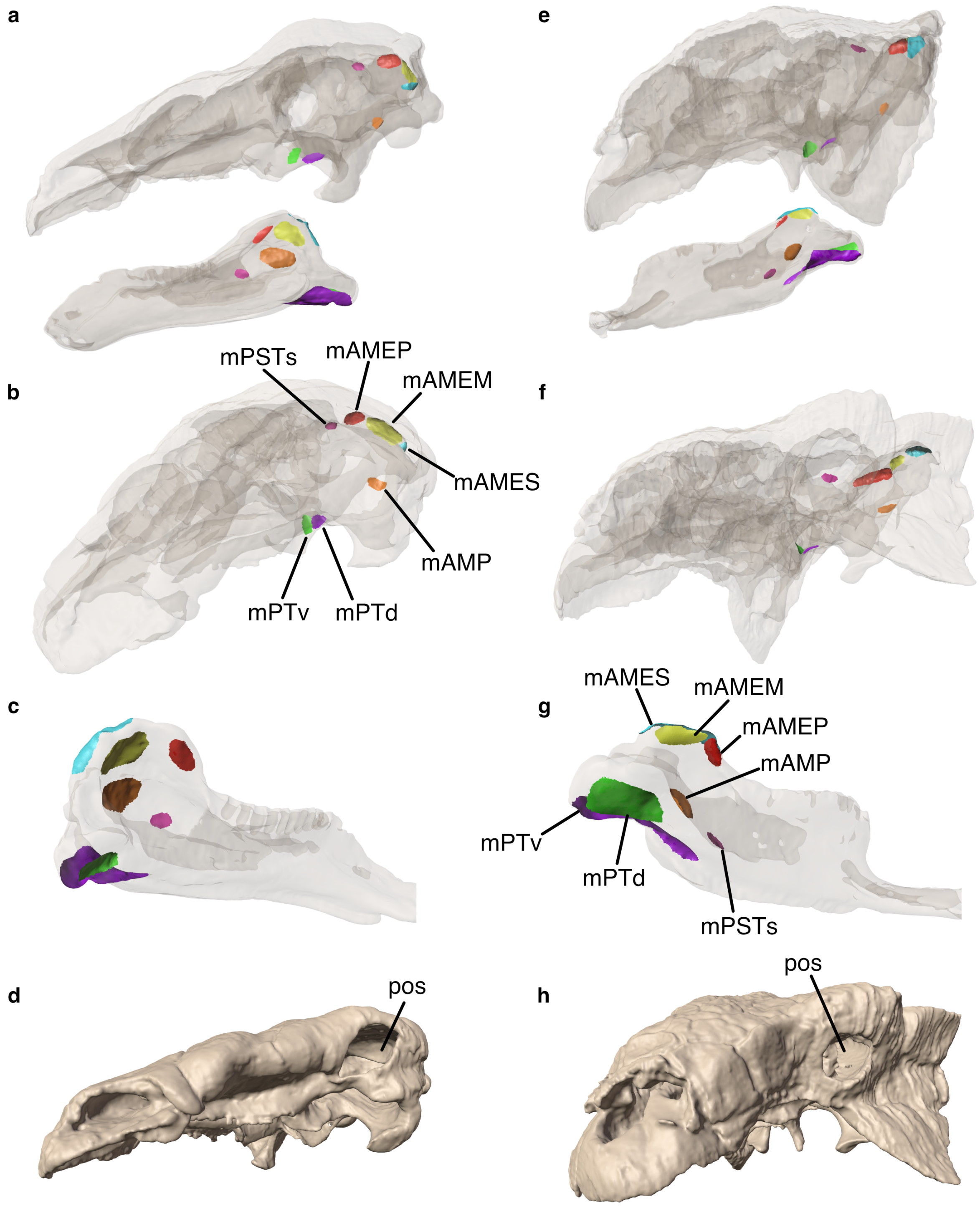

Figure S1. Origin and insertion sides of the jaw adductor muscles of *Panoplosaurus mirus* (**a**–**d**) and *Euoplocephalus tutus* (**e**–**h**). (**a, e**) Attachment sites on crania and mandibles in lateral view. (**b, f**) Detail of the muscle origin sites on the crania. (**c, g**) Detail of the muscle insertion sites on the mandibles. (**d, h**) Detail of the postocular shelf (pos).

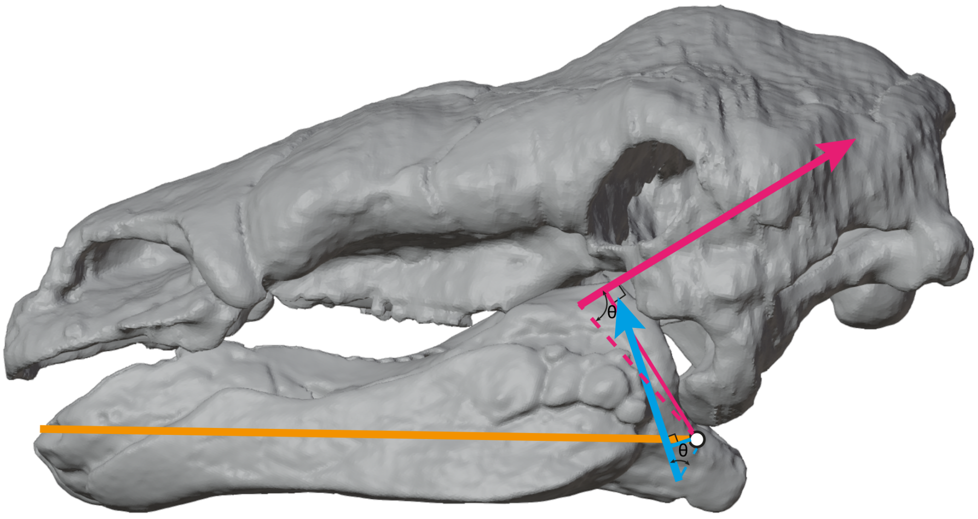

Figure S2. Lever system measurements used for bite force estimations, illustrated in the skull of *Panoplosaurus mirus* (ROM 1215). Solid coloured lines with arrows, lines of action for muscles. Dashed coloured lines, diagonal distance (d) from the mandibular muscle attachment sites to the jaw joint. White circle, the jaw joint fulcrum. q, the angle between the muscle line of action and d. Pink lines, lever system elements for m. adductor mandibulae externus profundus (mAMEP). Blue lines, lever system elements for m. pterygoideus dorsalis (mPTd). Orange line, the output lever (L_{out}) for biting at the muzzle.

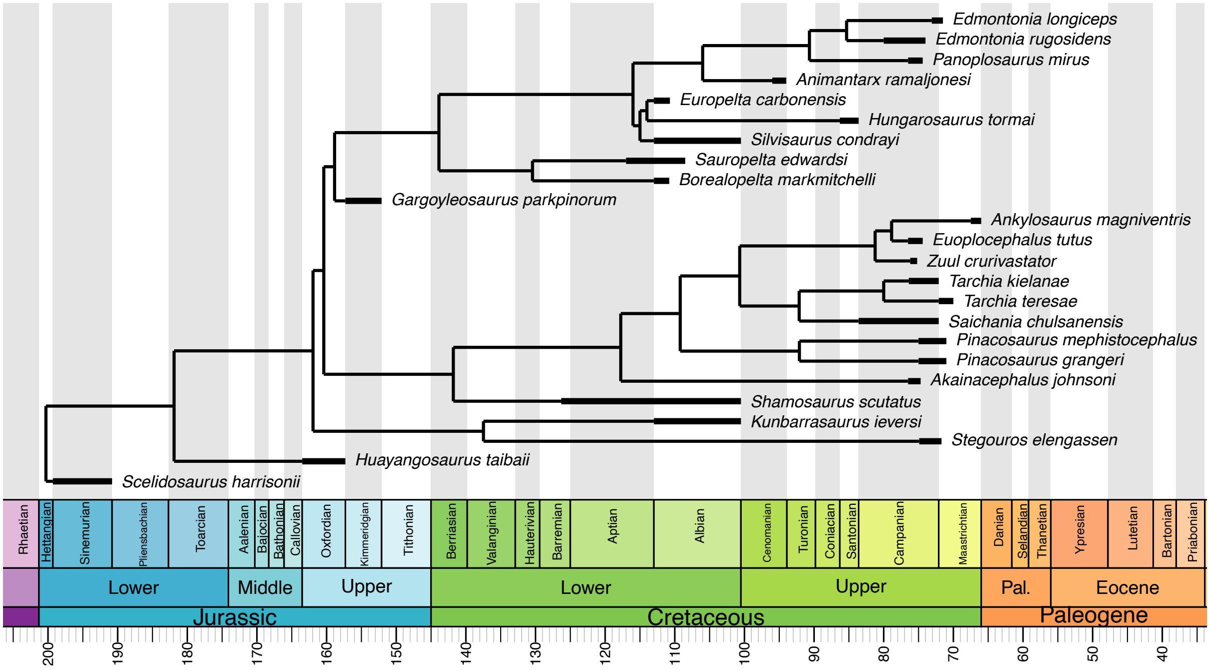

Figure S3. Time-calibrated phylogeny of Thyreophora used to reconstruct the evolution of mandibular mechanical advantage in ankylosaurs. The tree includes two outgroups of Ankylosauria: the early-diverging thyreophoran *Scelidosaurus* and the stegosaur *Huayangosaurus*.

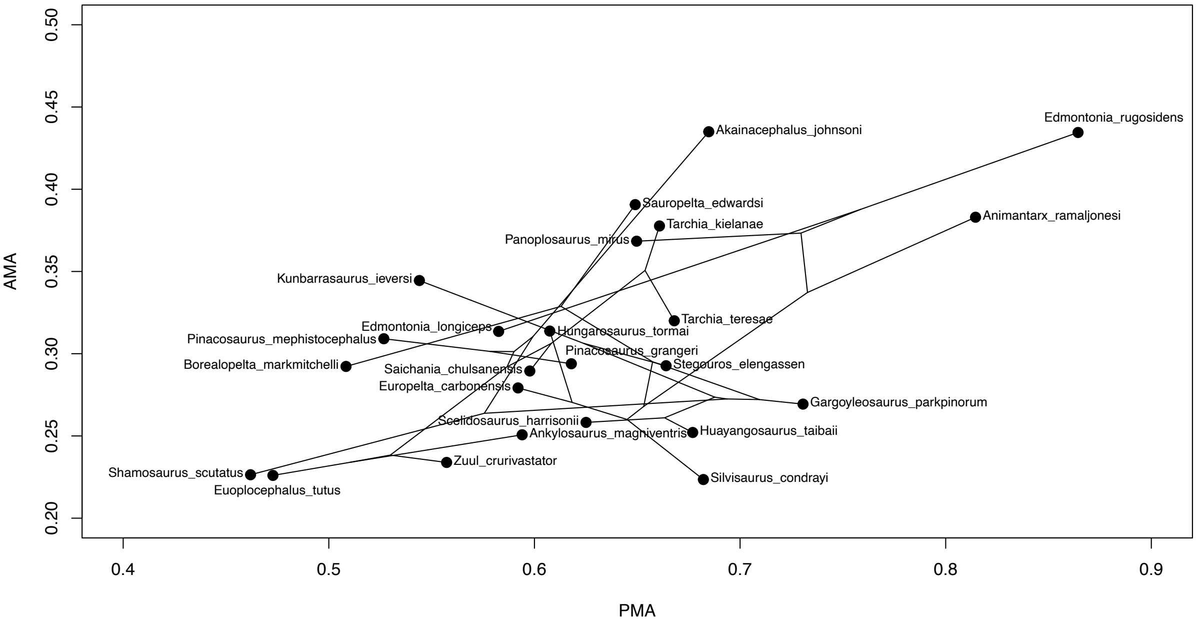

Figure S4. Basic phylomorphospace of anterior versus posterior mechanical advantage of Thyreophora, showing taxon names missing from Fig. 7c.

Table S1. Model properties of the FE models

FE model	Elements	Nodes	Mean EVOL (mm^3)
Euoplocephalus cranium	4126670	836216	1.340
Euoplocephalus cranium (no secondary palate)	3721591	753268	1.530
Euoplocephalus mandible	3435216	683236	0.210
Panoplosaurus cranium	4839557	972635	0.598
Panoplosaurus cranium (no secondary palate)	3836447	772468	0.762
Panoplosaurus mandible	3647871	725101	0.219

EVOL, element volume.

Table S2. Boundary condition setup of the FE models.

	Node count (cranium)	Node count (mandible)	Muscle forces *Euoplocephalus*	Muscle forces *Panoplosaurus* (scaled)
mAMEP	45	60	215.7	115.0
mAMEM	30	65	232.6	245.1
mAMES	50	75	307.3	133.8
mPTD	40	55	257.9	126.0
mPTV	45	90	597.8	474.2
mAMP	50	65	150.4	163.6
mPSTS	30	30	121.6	78.3
Anterior BP	8	6		
Muzzle BP	4	4		
Posterior BP	6	6		
Occipital condyle	6	N/A		
Paraoccipital process	10	N/A		
Quadrate/Glenoid	15	10		

Anterior BP, bite points for simulated biting at the anterior tooth row. Muzzle BP, bite points for simulated biting at the muzzle. Posterior BP, bite points for simulated biting at the posterior tooth row. All node counts except the ones for the occipital condyle and the paraoccipital process are unilateral.

Table S3. Ankylosaur species, specimens and image sources included in the mechanical advantage analysis.

Species	Specimen	Reference
Akainacephalus johnsoni	UMNH VP 20202	Weirsma & Irmis 2018
Animantarx ramaljonesi	CEUM 6228	Carpenter et al. 1999
Ankylosaurus magniventris	AMNH 5214	Carpenter 2004
Borealopelta markmitchelli	TMP 2011.033.0001	Brown et al. 2017
Edmontonia longiceps	NMC 8531	Sternberg 1928
Edmontonia rugosidens	USNM 11868	Carpenter 1990
Euoplocephalus tutus	AMNH 5405	Vickaryous & Russel 2003
Europelta carbonensis	AR-1/31	Kirkland et al. 2013
Gargoyleosaurus parkpinorum	DMNH 27726	Carpenter et al. 1998
Huayangosaurus taibaii	IVPP V6728	Sereno & Zhimin 1992
Hungarosaurus tormai	MTM 2007.25.1	Ősi & Makádi 2009
Kunbarrasaurus ieversi	QM F18101	Leahey et al. 2015
Panoplosaurus mirus	ROM 1215	Carpenter 1990
Pinacosaurus grangeri	IGM 100/1014	Hill et al. 2003
Pinacosaurus mephistocephalus	IMM 96BM3/1	Godefroit et al. 1999
Saichania chulsanensis	MPC 100/151	Maryańska 1977
Sauropelta edwardsi	AMNH 3035	Carpenter & Kirkland 1998
Scelidosaurus harrisonii	NHMUK R1111	Norman 2020
Shamosaurus scutatus	PIN N 3779/2-2	Tumanova 1987
Silvisaurus condrayi	KU 10296	Eaton 1960
Stegouros elengassen	CPAP-3165	Soto- Acuña et al. 2021
Tarchia kielanae	INBR 21004	Arbour et al. 2014
Tarchia teresae	PIN 3140/250	Ősi et al. 2017
Zuul crurivastator	ROM 75860	Arbour & Evans 2017

Table S4. Lower jaw lever arm measurements and mechanical advantage of ankylosaurs and outgroups.

Species	Li	PLo	ALo	PMA	AMA
Akainacephalus johnsoni	85.001	124.130	195.418	0.685	0.435
Animantarx ramaljonesi	126.760	155.628	330.987	0.815	0.383
Ankylosaurus magniventris	80.347	135.258	320.490	0.594	0.251
Edmontonia rugosidens	93.244	107.877	214.596	0.864	0.435
Euoplocephalus tutus	41.143	87.017	182.045	0.473	0.226
Europelta carbonensis	81.835	138.229	293.113	0.592	0.279
Gargoyleosaurus parkpinorum	49.246	67.404	182.827	0.731	0.269
Huayangosaurus taibaii	63.890	94.378	253.425	0.677	0.252
Hungarosaurus tormai	55.896	92.018	178.143	0.607	0.314
Kumbarrasaurus ieversi	60.715	111.599	176.244	0.544	0.344
Panoplosaurus mirus	79.371	122.165	215.448	0.650	0.368
Pinacosaurus grangeri	34.377	55.631	116.973	0.618	0.294
Pinacosaurus mephistocephalus	30.351	57.619	98.190	0.527	0.309
Saichania chulsanensis	53.121	88.869	183.499	0.598	0.289
Sauropelta edwardsi	96.430	148.578	246.846	0.649	0.391
Scelidosaurus harrisonii	55.847	89.341	216.268	0.625	0.258
Shamosaurus scutatus	57.158	123.735	252.461	0.462	0.226
Silvisaurus condrayi	71.458	104.752	319.732	0.682	0.223
Stegouros elengassen	63.682	95.904	217.574	0.664	0.293
Tarchia kielanae	61.143	92.527	161.896	0.661	0.378
Tarchia teresae	59.689	89.353	186.511	0.668	0.320
Zuul crurivastator	52.741	94.637	225.499	0.557	0.234
Borealopelta markmitchelli	71.784	141.206	245.620	0.508	0.292
Edmontonia longiceps	74.453	127.797	237.444	0.583	0.314

Li, in-lever. PLo, posterior out-lever. ALo, anterior out-lever. PMA, posterior mechanical advantage. AMA, anterior mechanical advantage.

References

Arbour, V. M., Currie, P. J. & Badamgarav, D. The ankylosaurid dinosaurs of the Upper Cretaceous Baruungoyot and Nemegt formations of Mongolia. *Zool. J. Linnean. Soc.* **172**, 631–652 (2014).

Arbour, V. M. & Evans, D. C. A new ankylosaurine dinosaur from the Judith River Formation of Montana, USA, based on an exceptional skeleton with soft tissue preservation. *Royal Society Open Science* **4**, 161086 (2017).

Brown, C. M. et al. An exceptionally preserved three-dimensional armored dinosaur reveals insights into coloration and Cretaceous predator-prey dynamics. *Curr. Biol.* **27**, 2514–2521 (2017).

Carpenter, K. In *Dinosaur Systematics: Perspectives and Approaches* (eds. Carpenter, K. & Currie, P. J.) 281–298 (Cambridge University Press, 1990).

Carpenter, K. & Kirkland, J. I. Review of Lower and Middle Cretaceous ankylosaurs from North America. *N. M. Mus. Nat. Hist. Sci. Bull.* **14**, 249–270 (1998).

Carpenter, K., Miles, C. & Cloward, K. Skull of a Jurassic ankylosaur (Dinosauria). *Nature* **393**, 782–783 (1998).

Carpenter, K., Kirkland, J. I., Burge, D. & Bird, J. In *Vertebrate Paleontology in Utah* (ed. Gillette, D. D.) 243–251 (Utah Geological Survey, 1999).

Carpenter, K. Redescription of *Ankylosaurus magniventris* Brown 1908 (Ankylosauridae) from the Upper Cretaceous of the Western Interior of North America. *Canadian Journal of Earth Sciences* **41**, 961–986 (2004).

Eaton, T. H. A new armored dinosaur from the Cretaceous of Kansas. *Univ. Kans. Paleontol. Contrib.*, **25**, 1–24 (1960).

Godefroit, P., Pereda-Suberbiola, X., Li, H. & Dong, Z.-M. A new species of the ankylosaurid dinosaur *Pinacosaurus* from the Late Cretaceous of Inner Mongolia (P. R. China). *Bull. Inst. Roy. Sci. Nat. Belg. Sci. Terre* **69**, 17–36 (1999).

Hill, R. V., Witmer, L. M. & Norell, M. A. A new specimen of *Pinacosaurus grangeri* (Dinosauria: Ornithischia) from the Late Cretaceous of Mongolia: ontogeny and phylogeny of ankylosaurs. *Am. Mus. Novit.* **3395**, 1–29 (2003).

Kirkland, J. I., Alcalá, L., Loewen, M.A., Espílez, E., Mampel, L. & Wiersma, J. P. The basal nodosaurid ankylosaur *Europelta carbonensis* n. gen., n. sp. from the Lower Cretaceous (lower Albian) Escucha Formation of northeastern Spain. *PLoS ONE* **8**, e80405 (2013).

Leahey, L. G., Molnar, R. E., Carpenter, K., Witmer, L. M. & Salisbury, S. W. Cranial

osteology of the ankylosaurian dinosaur formerly known as *Minmi* sp.(Ornithischia: Thyreophora) from the Lower Cretaceous Allaru Mudstone of Richmond, Queensland, Australia. *PeerJ* **3**, e1475 (2015).

Maryańska, T. Ankylosauridae (Dinosauria) from Mongolia. *Palaeontol. Pol.* **37**, 85–151 (1977).

Norman, D. B. *Scelidosaurus harrisonii* Owen, 1861 (Dinosauria: Ornithischia) from the Early Jurassic of Dorset, England: cranial anatomy. *Zool. J. Linn. Soc.* **188**, 1–81 (2020).

Ősi, A. & Makádi, L. New remains of *Hungarosaurus tormai* (Ankylosauria, Dinosauria) from the Upper Cretaceous of Hungary: Skeletal reconstruction and body mass estimation. *Palaontol. Z.* **83**, 227–245 (2009).

Ősi, A., Prondvai, E., Mallon, J. & Bodor, E. R. Diversity and convergences in the evolution of feeding adaptations in ankylosaurs (Dinosauria: Ornithischia). *Hist. Biol.* **29**, 539–570 (2017).

Sereno, P. C. & Zhimin, D. The skull of the basal stegosaur *Huayangosaurus taibaii*. *J. Vert. Paleontol.* **11**, 318–343 (1992).

Soto-Acuña, S., Vargas, A.O., Kaluza, J. *et al.* Bizarre tail weaponry in a transitional ankylosaur from subantarctic Chile. *Nature* **600**, 259–263 (2021).

Sternberg, C. M. A new armored dinosaur from the Edmonton Formation of Alberta. *R. Soc. Can. Trans.* **22**, 93–106 (1928).

Tumanova, T. A. The armored dinosaurs of Mongolia. *Joint Soviet Mongolian Paleontol. Exped. Trans.* **32**, 1–76 (1987).

Vickaryous, M. K. & Russell, A. P. A redescription of the skull of *Euoplocephalus tutus*(Archosauria: Ornithischia): a foundation for comparative and systematic studies of ankylosaurian dinosaurs. *Zool. J. Linn. Soc.* **137**, 157–186 (2003).

Wiersma, J. P. & Irmis, R. B. A new southern Laramidian ankylosaurid, *Akainacephalus johnsoni* gen. et sp. nov., from the Upper Campanian Kaiparowits formation of southern Utah. *PeerJ* **6**, e5016 (2018).
